# Leveraging technological advances to assess dyadic visual cognition during infancy in high- and low-resource settings

**DOI:** 10.3389/fpsyg.2024.1376552

**Published:** 2024-05-30

**Authors:** Prerna Aneja, Thomas Kinna, Jacob Newman, Saber Sami, Joe Cassidy, Jordan McCarthy, Madhuri Tiwari, Aarti Kumar, John P. Spencer

**Affiliations:** ^1^School of Psychology, University of East Anglia, Norwich, United Kingdom; ^2^School of Medicine, University of East Anglia, Norwich, United Kingdom; ^3^School of Pharmacy, University of East Anglia, Norwich, United Kingdom; ^4^IT and Computing, University of East Anglia, Norwich, United Kingdom; ^5^Community Empowerment Lab, Lucknow, India

**Keywords:** caregiver-infant dyads, cognitive development, infancy, eye-tracking, low-and middle-income countries (LMIC), visual attention

## Abstract

Caregiver-infant interactions shape infants' early visual experience; however, there is limited work from low-and middle-income countries (LMIC) in characterizing the visual cognitive dynamics of these interactions. Here, we present an innovative dyadic visual cognition pipeline using machine learning methods which captures, processes, and analyses the visual dynamics of caregiver-infant interactions across cultures. We undertake two studies to examine its application in both low (rural India) and high (urban UK) resource settings. Study 1 develops and validates the pipeline to process caregiver-infant interaction data captured using head-mounted cameras and eye-trackers. We use face detection and object recognition networks and validate these tools using 12 caregiver-infant dyads (4 dyads from a 6-month-old UK cohort, 4 dyads from a 6-month-old India cohort, and 4 dyads from a 9-month-old India cohort). Results show robust and accurate face and toy detection, as well as a high percent agreement between processed and manually coded dyadic interactions. Study 2 applied the pipeline to a larger data set (25 6-month-olds from the UK, 31 6-month-olds from India, and 37 9-month-olds from India) with the aim of comparing the visual dynamics of caregiver-infant interaction across the two cultural settings. Results show remarkable correspondence between key measures of visual exploration across cultures, including longer mean look durations during infant-led joint attention episodes. In addition, we found several differences across cultures. Most notably, infants in the UK had a higher proportion of infant-led joint attention episodes consistent with a child-centered view of parenting common in western middle-class families. In summary, the pipeline we report provides an objective assessment tool to quantify the visual dynamics of caregiver-infant interaction across high- and low-resource settings.

## 1 Introduction

Visual exploration is one of the early building blocks for learning in infancy. In real-world settings, infants' visual experience is complex with often cluttered environments. Infants typically explore these environments with social partners who have their own visual attention abilities and preferences. Critically, early interactions between infants and their caregivers influence early learning and are important for shaping developmental outcomes (Tamis-LeMonda et al., 2004; Evans and Porter, 2009; Yu et al., 2019). Therefore, understanding the nature of social interactions and developing objective measures to assess these interactions is critical.

Previous anthropological and psychological research on parent-infant interaction has used a third-person view to capture interactions in low-resource settings. These observations have been traditionally coded in real-time by a trained observer. However, as technologies have advanced, videotaping interactions has become the norm with analyses annotating the videos frame by frame (Abels, 2020; Schmidt et al., 2023). There are two main issues with human coding of interactions: (1) a limited amount of participants' data can be collected because of the time and effort that goes into frame-by-frame coding, and (2) the third-person perspective offers only limited insight into parents' and infants' own visual experiences. The current study uses technological advances to create a pipeline to collect, process, and analyse parent-infant interaction data using open-source and freely accessible algorithms.

This paper builds on recent work that has quantified parent-infant interactions using innovative technologies (Yoshida and Smith, 2008; Aslin, 2009; Smith et al., 2011, 2015; Yu and Smith, 2012). One such technology is the head-mounted eye-tracker. Unlike older technologies such as hand-held cameras, head-mounted cameras and eye trackers provide a closer approximation of what individuals see and how they deploy their attention. Using these technologies, researchers have found that the visual dynamics of parent and infant are very different compared to a third-person's view. For example, Smith et al. (2011) recorded a ten-minute toy play session between parents and their 17- to 19- month-old infants using head-mounted cameras and eye-trackers. Results suggested that the infants' first-person view was highly selective with one dominating object (toy) in view. Critically, this object blocked the view of other objects around the infant. In contrast, the parents' first-person view was broader and more stable. Parents tended to shift their gaze between visual targets (toys, hands, and infant's face) rapidly with all objects equally in view. Interestingly, infants' momentary visual experience included more hands manipulating objects—their own and the parents—than parents' momentary visual experience (see also Yoshida and Smith, 2008).

A key explanation for the difference in infants' visual experience comes from the fact that infants' bodily movements such as turning heads or reaching for a toy both have a major influence on visual dynamics (Schneiberg et al., 2002; Yoshida and Smith, 2008; Smith et al., 2011). Parents also played a role in selecting targets for the infant's view (Xu et al., 2011). Franchak et al. (2011) noted that 14-month-old infants frequently fixated on caregivers' hands and bodies instead of caregivers' faces. Moreover, infants were more likely to look at the mother's face if the mother was sitting down at the infant's eye level versus standing upright. In summary, head-mounted eye trackers and cameras enable one to capture first-person visual experiences which can systematically differ from a third-person perspective (Aslin, 2009) yielding insights which are not always intuitive relative to the adult third-person view (Yurovsky et al., 2013).

The goal of the present study was to assess whether head-mounted eye trackers could be used to meaningfully examine dyadic infant-caregiver interactions within global, low-resource settings, and generate objective and quantifiable measures. Much of the research in psychology comes from Western, high resource societies. In principle, however, portable eye-tracking technologies should be useable even in rural low- and middle-income countries (LMIC) where research infrastructure is minimal. To our knowledge, no study has deployed head-mounted eye-trackers and head cameras in low-resource settings. Thus, here we used this technology to extract measures of visual exploration and visual cognition during parent-infant interaction in Norwich, UK (urban UK) and Shivgarh, India (rural India). This allowed us to develop a processing pipeline that generalized across data sets, extracting common measures across socio-cultural contexts.

We focused on using open-source machine learning algorithms with the goal of avoiding laborious frame-by-frame hand coding. This enabled us to process large data sets in an objective manner that was fully transferable across cultures. In particular, we used machine learning algorithms to quantify how long each member of a dyad looked at each object and how often they looked at the face of the social partner. From these data, we then calculated key measures such as how long they sustained attention on objects and faces, and who is leading and who is following each attentional episode.

The paper proceeds as follows. In Study 1, we describe how we developed and validated a new pipeline for processing dyadic data from caregivers and infants. To probe the effectiveness of this pipeline, we applied it to a subset of data from two parallel studies—one conducted in an urban UK setting and one from rural India. In Study 2, we then applied this pipeline to the full samples from both studies, revealing novel insights into how visual dynamics unfold across two socio-cultural contexts.

## 2 Study 1: developing a pipeline for the analysis of dyadic visual cognition across cultures

The goal of this initial study was to develop a pipeline for the analysis of real-world dyadic interactions between a caregiver and infant that generalizes across cultures. In the sections that follow, we introduce the data collection methods used in both the urban UK and rural India settings. Next, we discuss the machine learning tools used to process the data set, including a face-detection network and an object recognition network. For each machine learning approach, we discuss how the networks were trained, validated, and optimized. We then describe the full processing pipeline, including a discussion of a toolkit for analyzing the resultant time series data. Next, we apply this pipeline to a subset of 12 infant-caregiver dyads: 4 from a 6-month-old UK cohort, 4 from a 6-month-old India cohort, and 4 from a 9-month-old India cohort. Here, we validate the pipeline performance by quantifying the accuracy of the resultant data set relative to hand-coded data. This work sets the stage for a larger cross-cultural comparison of dyadic interactions which is presented in Study 2.

## 3 Data collection methods

### 3.1 Participants

We present data from four parent-infant dyads from the UK: infants aged 6 months ±15 days (3 females, *M* = 5.46 months, *SD* = 0.84 months). All families were recruited by the Developmental Dynamics Lab at the University of East Anglia for a longitudinal project on early brain development. Inclusion criteria for dyads included (1) normal or corrected-to-normal vision; (2) uncomplicated single birth between 37 and 42 weeks; (3) no reports of alcohol or drug illicit use during pregnancy; (4) no pre-existing neurological conditions or major head injury; (5) no familial history of major depressive or psychiatric illness confirmed during the parental interview during enrolment. Parents were informed of the experiment's aim and procedure, and written consent was obtained. Remuneration comprised of 20 pounds, travel expenses, a t-shirt and a toy for each participant.

The Indian sample was recruited with the help of the Community Empowerment Lab (CEL) which works in the rural area of Shivgarh in the state of Uttar Pradesh. Uttar Pradesh has one of the highest infant mortality rates in India with 60 deaths per 1000 live births for children under five years of age (see National Family Health Survey report 2019–2021). The current study reports data from eight dyads: four infants were aged 6 months ±15 days (3 females, *M* = 6.06 months, *SD* = 0.23 months) and four infants were aged 9-month ±15 days (3 females, *M* = 9.02 months, *SD* = 0.42 months). All the infants were full-term and typically developing.

### 3.2 Materials

#### 3.2.1 Mobile eye-tracking

Caregivers' eye movements and caregivers' and infants' visual fields were recorded using light-weight (36gms) mobile eye-trackers developed by Pupil Labs (Kassner et al., 2014). The eye tracker was used with the software Pupil Capture (versions 0.09 to 0.9.15). The eye-tracker has an infrared eye camera, placed close to the eye, that recorded monocular pupil and corneal reflections at a resolution of 640 × 480 pixels and a sampling rate of 120 Hz. The world camera was captured at 30 Hz at a resolution of 1,280 × 720 pixels. The eye-tracker and head-mounted cameras were connected to mobile phones. For data collection in Shivgarh, we used Nexus 5XN4F2T mobile phones. For the UK, we used Google Pixel 2 mobile phones. The mobile phones captured data using the Pupil Mobile app. These mobile phones were in turn connected to a laptop (HP laptop in India and Mac laptop in the UK) through a WiFi network to enable the simultaneous streaming of the video. This allowed the experimenter to monitor the session remotely. The parent wore the head-mounted eye-trackers like glasses with a nose-piece placed on the nose. The infant's head-mounted camera was embedded into a headband for comfort as well as to avoid slippage. In line with previous research using head-mounted cameras, we placed the camera low on the infant's forehead (Smith et al., 2011). We did not use the eye camera with infants as they did not like the camera placed close to their eyes.

### 3.3 Stimuli

**Figure 1:**
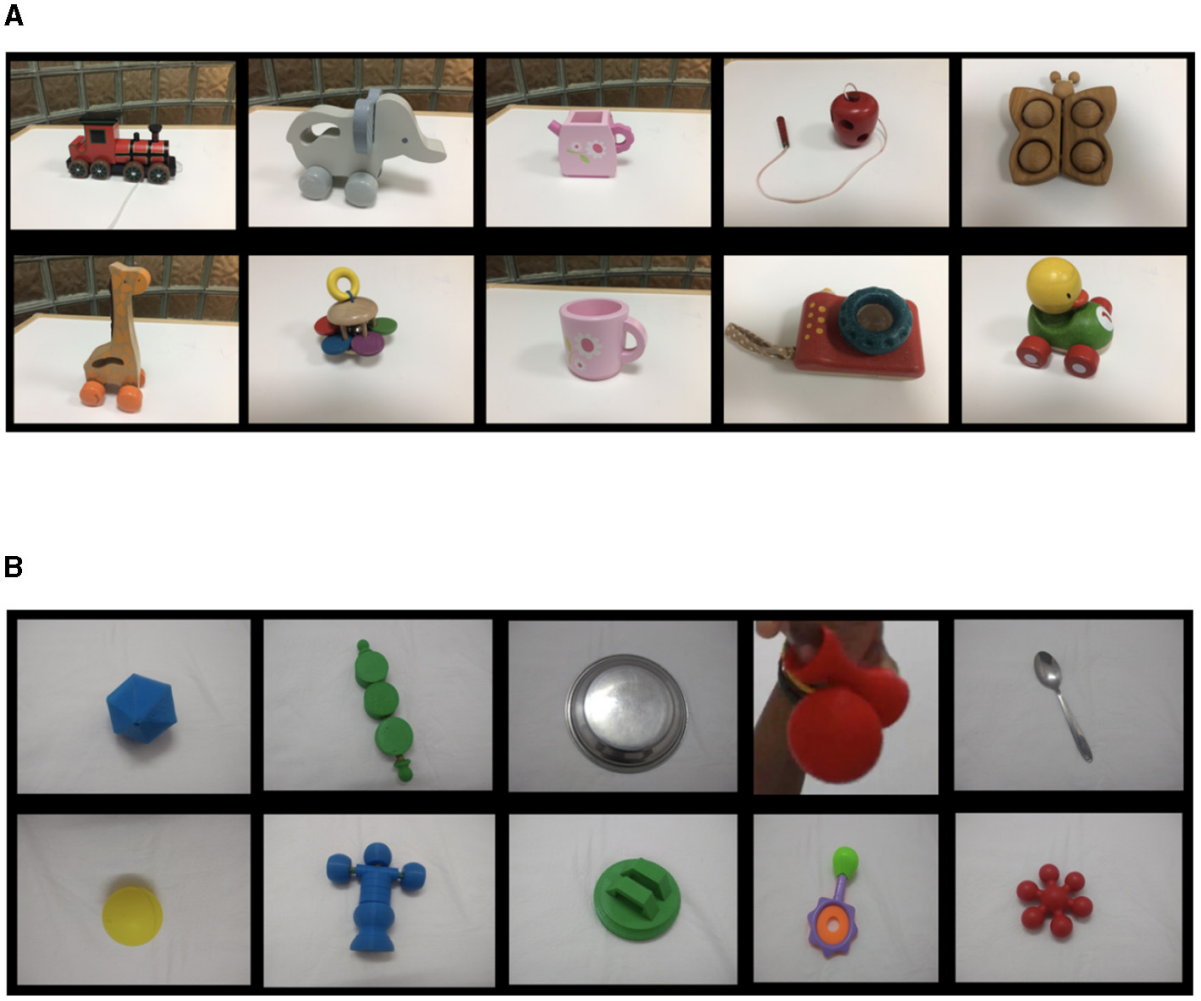
Set of toys used in the UK **(A)** and India **(B)**. **(A)** Top (left to right) Train, Elephant, Kettle, Apple, Butterfly. Bottom (left to right) Giraffe, Rattle, Cup, Camera, Duck. **(B)** For some objects, we made up names to create labels to train them in the YOLO algorithm. Top (left to right) Blue ball, Candy, Plate, Glow, Spoon. Bottom (left to right) Yellow ball, Man, Green, Rattle, Puzzle.

Ten toys were organized into two sets with each set containing five toys in the UK. The toys include utensils, animals, and/or different shaped blocks of single main color (see Figure 1A). If and when the toys broke during data collection, they were replaced by another toy. For example, a toy apple in the UK was replaced with a toy pear.

Similarly, ten toys were used in India including objects used as toys in the local community (e.g., plate, spoon), familiar toys (e.g., rattle, ball) and novel toys (see Figure 1B). If the toys broke during data collection, we either replaced them with another toy or repaired them with the exception of one toy (GLOW) as parents and infants continued to play with the dismantled toy.

It is important to acknowledge that our decision to use different toys across contexts was motivated by two factors. First, in India, preliminary discussions with families revealed that most infants played with common household objects (e.g., plate, spoon) rather than store-bought toys. Thus, we included a subset of these items to make the toys culturally relevant. Second, the study in India began prior to the study in the UK. At that time, machine learning networks were particularly sensitive to color. Consequently, our collaborators suggested we use novel toys with a homogeneous color. By the time the UK study started, it was clear that newer machine learning methods did not require homogeneous color. Thus, we relaxed this criterion when selecting culturally-relevant toys for the UK sample.

### 3.4 Setup UK

Trained researchers visited the participant's home at a time when the parents confirmed that the infants were usually awake and fed. After obtaining consent from the parent, the eye trackers and tripod cameras were set up in an area where the parents typically played with the infant. Infants wore a vest to which the mobiles were attached at the back for freedom of movement. If the infant was on a boppy pillow, then the cable and phone were left on the side. Parents were briefed that they were free to pick up their infants and move the phone/cable as desired. The experimenter adjusted the scene and eye cameras when necessary. Parents wore a lab coat with a pocket or velcro at the back of the lab coat to attach the phone for freedom of movement. Two tripod-mounted cameras captured the play session from a third-person view.

### 3.5 Setup India

Due to infrastructural and technological constraints (e.g., lack of electricity), the parent-infant interaction study took place in several rooms set up as an open laboratory space in a palace where we could bring in a generator for power cuts. Before the sessions began, families toured the laboratory while all procedures were explained to them. Families were shown the equipment, explained its function and were given the opportunity to ask any questions. They were, then, seated in a common playroom where consent was given.

**Figure 2:**
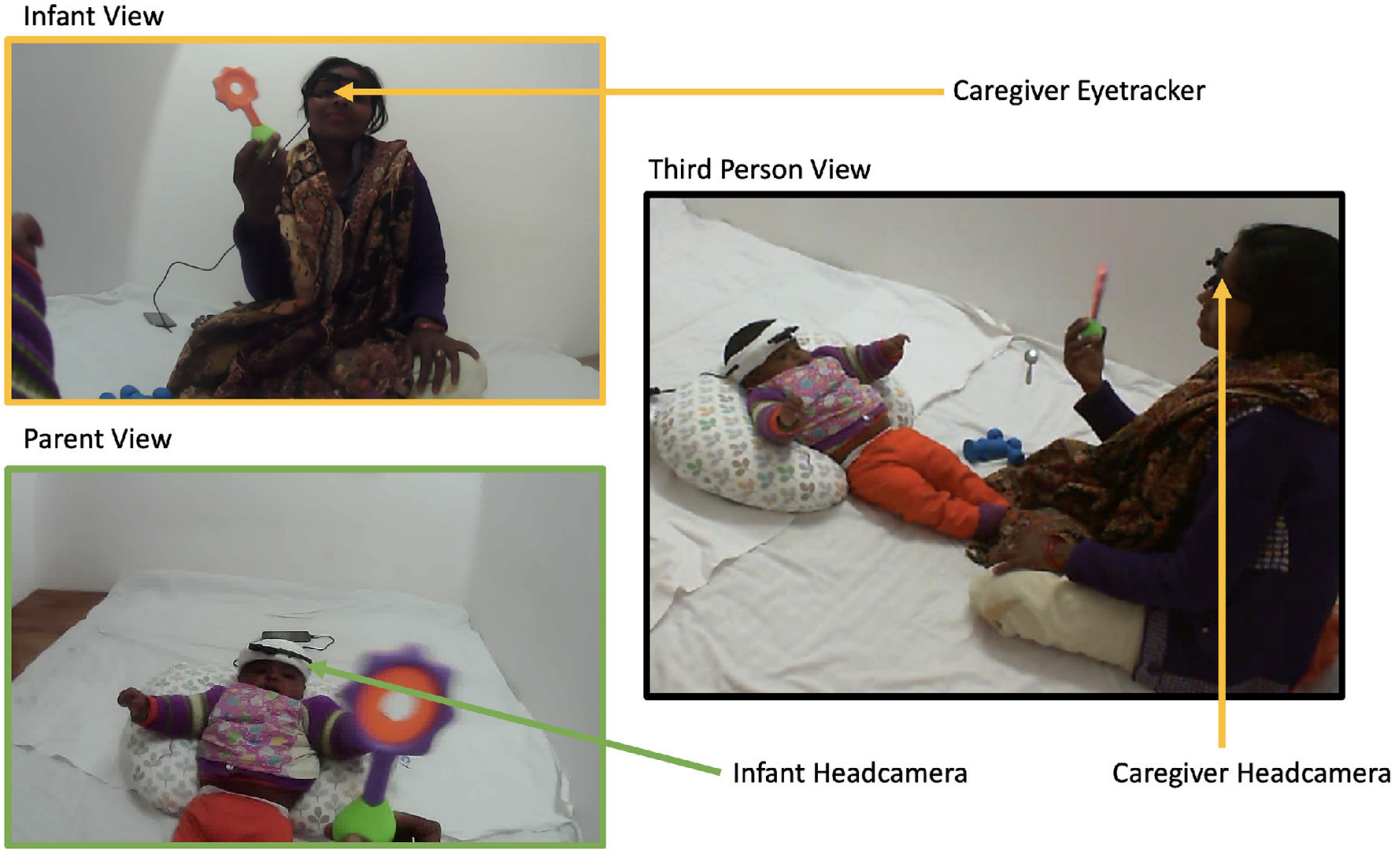
Caregiver-Infant interaction setup in Shivgarh, India. Caregiver-infant dyads played together with a set of toys in a naturalistic setting. Each wore head-mounted cameras to collect egocentric video and the caregivers also wore eye-tracker to track gaze positions **(left)**. A stationary camera is recorded from a third-person perspective **(right)**.

The parent-infant interaction room consisted of a mattress on the floor as a play area. Two cameras were placed on opposite walls to record parent-infant interaction from a third-person view. Figure 2 shows an example of the setup for the head-mounted eye-trackers and cameras in India. The set-up for eye-trackers was the same as in the UK with the exception that caregivers in India did not wear lab coats and hence mobile phones were placed next to them. Mothers felt uncomfortable wearing white lab coats because white is associated with widowhood, and mothers' felt uncomfortable wearing a lab coat that was very different from their usual attire. Caregivers were briefed that they were free to move around while holding their mobile phones if needed. Culturally, sitting on the floor, cross-legged or in a squatting position is an everyday practice. The caregivers hardly ever moved around the room or expressed a desire to move around the room with their infants.

### 3.6 Procedure

There were two experimenters in the room. In India, one of the experimenters was a staff member from the Community Empowerment Lab (CEL) and one was from the local community. Experimenter 1 helped the caregiver wear the head-mounted eye-tracker. Prior to calibrating the eye-tracker, Experimenter 1 would ask the parent to follow their finger (left, right, top, bottom) to make sure that the pupil was captured properly and to ensure that it was visible in the world camera. We used a minimum of nine calibration points to calibrate the eye-tracker using Pupil Capture software (for more information see https://docs.pupil-labs.com/core/). During calibration, the caregiver fixated on the calibration marker while keeping the head stationary, and the experimenter moved the marker around while staying within the participant's visual field (about 1.5–2 m away). The experimenter moved the calibration marker in such a way that it covered the 2D screen that was monitored by experimenter 2. Following the calibration, one of the two experimenters distracted the infant with a toy while the other experimenter placed the headband on the child's head. Experimenter 1 moved the toy in different directions (top, down, left, right) while Experimenter 2 adjusted the angle of the camera to ensure that the toy was in the infant's field of view when they moved their heads in different directions.

Once the cameras were set up, we started the infants' head camera. The experimenter placed the toys near the dyads within reach. Caregivers were instructed to play as they usually would with their infants. The experimenter placed a movie clapperboard between the parent and the infants' head camera such that it was visible on both the recordings and clapped it three times to synchronize the onset of the play session. Both the experimenters left the room (India) or moved to a corner of the room (UK) and checked the play session as it streamed live on the laptop for any issues (such as removing the head camera, technical issues, software errors, and so on). The play sessions were recorded for approximately 10 minutes.

## 4 Machine learning methods

The use of CNNs (convolutional neural networks) has gained popularity due to many successful vision-related applications including face detection (Zhang et al., 2016), object recognition (Redmon et al., 2016) and image-based diagnostic applications such as detecting anomalies in X-ray and MRI images (Yu et al., 2018). Here, we used several specific CNN tools to objectively detect the presence of faces and toys in the video data collected from the mothers' eye-tracker and the infants' head cameras.

### 4.1 Multi-cascade convolutional neural network for face detection

We used a publicly available multi-cascade convolutional neural network (MTCNN) focusing on the MTCNN face detection network built by Zhang et al. (2016). MTCNN is a fast, efficient and robust face detection algorithm (Zhang et al., 2020b) built to account for various illuminations and occlusion in real-world environments. MTCNN has recently been used by Long et al. (2022) with developmental data and resulted in good accuracy for face detection. Moreover, it can be used on both static images as well as videos.

We set up the MTCNN environment using Anaconda Navigator. We used the Tensorflow implementation of the MTCNN algorithm available at https://github.com/ipazc/mtcnn.

To test the accuracy of the MTCNN algorithm, we extracted 10 images from 10 dyads (5 from the parent head camera and 5 from the infant head camera): 80 face images and 20 non-face images. Half of the images were of infant faces and the other half of caregivers' faces. Images with various orientations, lighting and distance were selected. All the images were hand-labeled for faces using ImageJ, an open-source image processing and analyzing software (Schneider et al., 2012) yielding a .ROI file for each image containing the coordinates of the labeled box containing the face [the “Ground Truth” (GT) data set]. Faces were classified as present if at least half of the face was visible.

Sometimes, poor lighting conditions can affect image contrast, reducing the accuracy of face or object detection. We applied contrast limited adaptive histogram equalization (CLAHE) to images prior to detection. CLAHE increases image contrast, whilst avoiding the over-amplification of noise that is sometimes associated with adaptive histogram amplification (see Zuiderveld, 1994).

To evaluate the performance of the MTCNN face detector on our test data sets, we used Intersection Over Union (IoU) (see Padilla et al., 2021). IoU quantifies the extent of overlap between the GT and the prediction (the greater the overlap, the greater the IoU). To evaluate the precision and recall of the detections, it is necessary to establish an IoU threshold. The larger the threshold, the larger the IoU, and therefore, the overlap required for a “hit.” Using the threshold, the evaluation metric will classify each detection as:

1. True Positive, if IoU between the GT and prediction is greater than the IoU threshold;
2. False positive, if the IoU between GT and prediction is less than the IoU threshold or if there is a prediction without an associated GT;
3. False Negative, if the GT has a face yet there is no associated prediction;
4. True Negative, if the frame has no GT and no prediction.

We ran two evaluation metrics for each cohort (India, UK), one with CLAHE and another without CLAHE, using an IoU threshold of 0.50. To assess the performance, we calculated precision and recall. Precision, is the total number of true positives divided by the total number of true positives and false positives, in other words, it is the correctly identified faces out of all the identifications. Recall is the ratio of true positives and total GT positives, i.e., true positives divided by the sum of true positives and false negatives. The trade-off between precision and recall performance can be manipulated by adjusting the IoU threshold. Last, we calculated the Average Precision (AP): the precision averaged across all unique recalls.

For the UK cohort, without CLAHE, the detector retrieved 55.34% of the total ground truths. In contrast, with CLAHE, the detector retrieved 62% of total ground truths. For the India cohort, the detector retrieved 54.71% of total ground truths both with and without CLAHE. Based on these evaluation metrics, we decided to use CLAHE for both India and UK cohorts.

To improve detection performance, we visualized the MTCNN predictions using an open-source, video coding software called BORIS—Behavioral Observation Research Interactive Software (Friard and Gamba, 2016). After reviewing the MTCNN predictions, we decided to filter the data through different minimal bounding box sizes and confidence thresholds (ranging from 60%–80%) until we found the best precision. The best filter parameters that suited both cohorts were a minimal bounding box size of 5% of the image size, a confidence threshold of 70%, and an IOU threshold of 0.30. Implementation of the filter parameters increased the average precision for the UK cohort by 7.18% and for the India cohort by 3.45%.

To increase the accuracy further, we also filtered by asymmetry, that is, if an edge of the bounding box was five times longer than the adjacent side. Finally, we removed duplicate predictions, that is, if there were two bounding boxes for the same item (e.g., parent face) on the same frame. Removal was prioritized based on the size of the bounding box (i.e., the largest bounding box was kept) and secondarily based on confidence.

### 4.2 You only look once for object recognition

You Only Look Once (YOLO) is a state-of-the-art object detection algorithm developed by Redmon et al. (2016). It uses Convolutional Neural Networks (CNNs) to detect objects with better than real-time performance, typically processing in excess of 40 frames per second. Given the speed of detection, it is the preferred approach for a number of real-life applications, including identifying people and traffic signals on busy roads for autonomous driving applications (Pouyanfar et al., 2019; Masmoudi et al., 2021; Boukerche and Hou, 2022).

Here, we use YOLOv5 available at https://github.com/ultralytics/yolov5 (see also Jocher et al., 2022). The YOLO algorithm overlays a grid on the chosen image and makes a prediction for each cell in the grid. Predictions, bounding boxes, and confidence values are set for all cells, regardless of whether there are any salient objects or specified targets within them. Then, YOLO expands the bounding boxes by an amount proportional to the confidence of each prediction. Finaly, YOLO creates a map with multiple bounding boxes ranked by their confidence value, which serves to identify where the objects are located in the image.

To detect what the objects are, the algorithm predicts the probability that the image contains an object of a given class (e.g., plant) at each specific location. Predictions are conditional at this stage because they do not specify the presence of an object. Instead, they set the condition that “if” there is an object within the cell, that object will be of the given class. Next, YOLO multiplies the conditional probability with the objects' confidence value, resulting in bounding boxes weighted by their actual probabilities of containing the desired object(s). Then, it discards predictions with lower confidence values.

We trained separate YOLO models for the UK and India dyads. To train the models for the 11 UK toys and 10 India toys (and an additional class for a mobile phone), we manually labeled 1,295 frames sampled from 28 UK dyads from our larger data set (see Study 2), and 1238 frames sampled from 113 India dyads from our larger data set (see Study 2). The frames were extracted from both the parent and infants' head-mounted cameras. The initial selection of frames was random, however, we then inspected the selection and added additional frames to make sure that the data contained a good variety of different scales and orientations of each toy.

Frames were labeled by trained research assistants using a software package called LabelImg (Tzutalin, 2015). From each cohort's training data set, we extracted 128 (UK) and 117 (India) images to create a validation dataset, leaving us with 1,167 training images for the UK model and 1,120 training images for the India model. The accuracy of the models was assessed using precision, recall and mean average precision (mAP). All models were run at a threshold of 0.50 for Intersection Over Union (IoU).

The training, validation and testing was undertaken at the University of East Anglia's High-Performance Computing Cluster (HPC) which allowed for the processing of multiple dyads in parallel. The source code for YOLOv5 was downloaded from GitHub. The YOLO toolbox contains separate Python scripts for training and testing. We used a form of transfer learning, meaning that a pre-trained model is first loaded, and then the weights in that model are fine-tuned for the specific task at hand. The pre-trained model will already have been trained using many hundreds of thousands of images, drastically reducing the amount of new data required to adapt the model.

Training was conducted over a maximum of 250 epochs. During each epoch, small quantities of data (batches) are passed through the network and the weights and biases at each node of the network are tuned to minimize the network's loss (the difference between the predictions and the ground truth labels). We used the default batch size of 12 images. YOLO uses an early stopping criteria, causing the training to stop automatically if the loss stops reducing significantly between epochs. The training process produced two models: The model produced by the final epoch and the best model, as identified by the model with the lowest loss. We use the best model for processing our validation and test data.

#### 4.2.1 YOLO model evaluation UK

In an initial model, we used 1,167 images in the training data set and 128 images with 352 labels in the validation data set. A mAP of 0.832 was achieved across all toys.

Next, we incrementally increased the number of frames for the poorly performing toys to see if this improved the accuracy. We started by adding 20 toy elephant labels to the training dataset. This improved performance with a mAP of 0.858. Our final model included an updated training data set with 60 new labels (20 elephants, 20 giraffes and 20 cameras) as well as the updated validation set with 364 labels. As shown in Table 1, the overall mAP across toys was 0.856 with 96.2% precision and 75.2% recall.

**Table 1:** Performance of object detector for UK and India, at IoU threshold 0.50.

**Model 4 UK**				
**Class**	**label**	**Precision**	**Recall**	**mAP@.5**
All	364	0.962	0.752	0.856
TOY_APP	24	1	0.762	0.841
TOY_BUT	35	1	0.667	0.748
TOY_CAM	27	1	0.739	0.841
TOY_CUP	35	1	0.785	0.933
TOY_ELE	32	0.999	0.688	0.789
TOY_GIR	37	0.797	0.568	0.684
TOY_DUC	35	1	0.841	0.925
TOY_GRE	27	0.993	0.815	0.92
TOY_KET	45	1	0.721	0.913
TOY_RAT	31	0.89	0.871	0.927
TOY_TRA	36	0.908	0.82	0.9
**Model 3 India**				
**Class**	**Label**	**Precision**	**Recall**	**mAP@.5**
All	324	0.935	0.833	0.898
TOY_BBAL	21	0.942	1	0.95
TOY_CAN	38	0.969	0.835	0.943
TOY_GRE	5	0.927	0.8	0.8
TOY_MAN	46	0.918	0.87	0.91
TOY_PLA	49	0.949	0.767	0.81
TOY_PUZ	4	0.839	1	0.995
TOY_RAT	6	1	0.729	0.995
TOY_SPO	49	0.975	0.633	0.747
TOY_YEL	8	0.96	1	0.995
TOY_GLO	71	1	0.859	0.925
MOBILE	27	0.804	0.667	0.803

Final UK model included 1,227 images in the training dataset, and 128 images and 364 labels in the validation dataset. Final India model included 1,120 images in the training dataset, and 117 images and 364 labels in the validation dataset. CLAHE histogram equalizer was added to the India model. TOY_RED, TOY_MIX and TOY_GLO were combined into TOY_GLO.

For UK class, APP, Apple; BUT, Butterfly; CAM, Camera; CUP, Cup, ELE, Elephant, GIR, Giraffe, DUC, Duck; GRE, Green; Ket, Kettle; RAT, Rattle; TRA, Train. For India class, BBAL, Blue Ball; CAN, Candy; GRE, Green; MAN, Man; PLA, Plate; PUZ, Puzzle; RAT, Rattle; RED, Red; SPO, Spoon; YEL, Yellow Ball; MIX, Mix; GLO, Glow.

#### 4.2.2 YOLO model evaluation India

One of the toys in India broke during the study, and caregivers and their infants started using them as novel toys so we decided not to change them. Therefore, during labeling for the India training dataset, we labeled the TOY_GLO as a whole and its dismantled parts as TOY_RED and TOY_MIX. In our initial India model, we achieved an overall mAP across toys of 0.839. However, labels such as TOY_RED, TOY_MIX and TOY_GLO showed a relatively poor performance. Therefore, to enhance the results for these labels, we added CLHAE image processing to our training data set (previously used in MTCNN), but this did not enhance performance.

For our next model, we decided to combine labels of TOY_GLO, TOY_MIX and TOY_RED into one label, namely, TOY_GLO, given that they were all dismantled parts of the same toy. The final model showed an improved accuracy for object detection with an overall mAP of 0.898 across all toys (see Table 1).

## 5 Implementation of the processing pipeline

**Figure 3:**
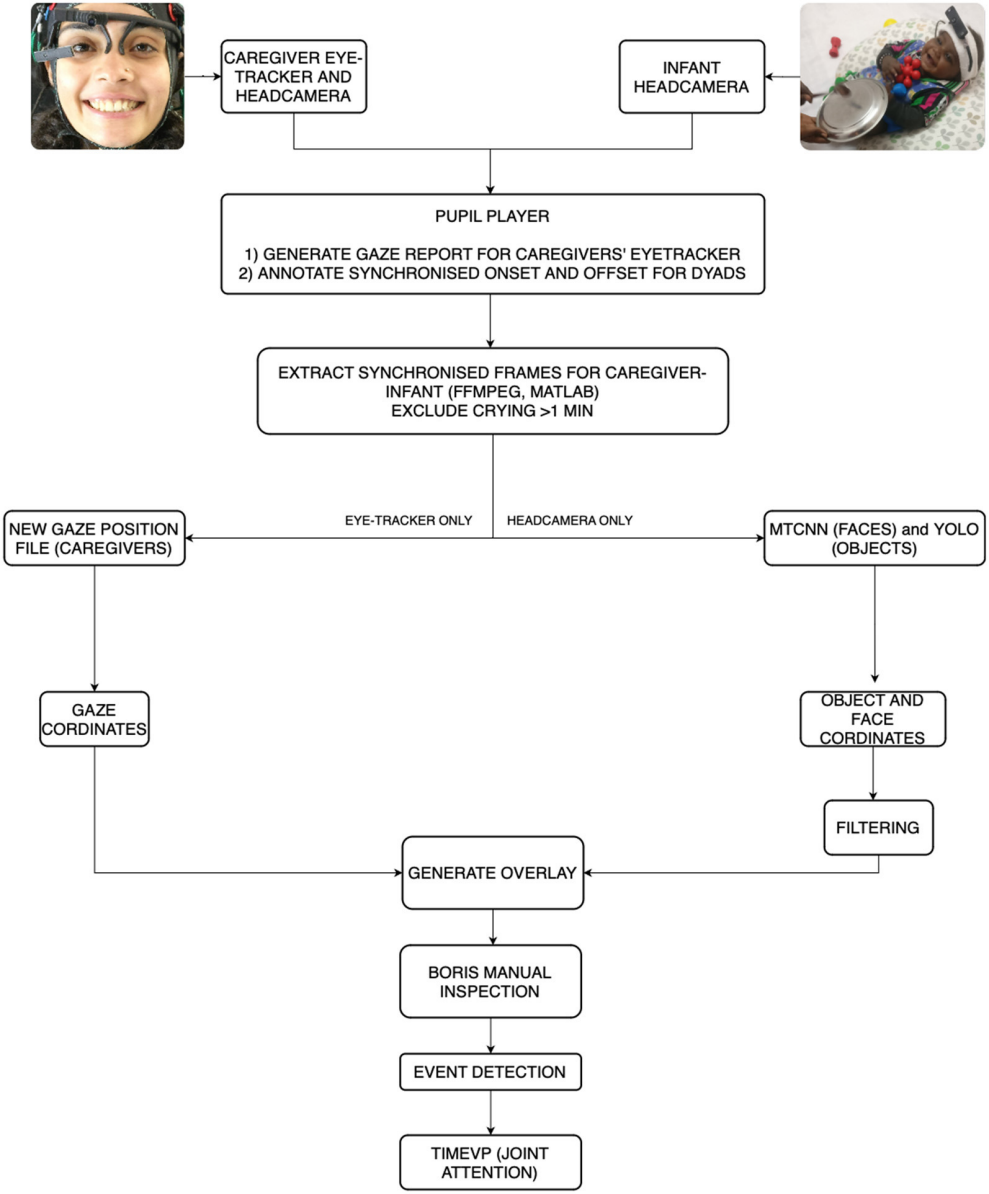
A flow diagram demonstrating the steps of the pipeline.

Now that the CNNs for faces and objects were trained and validated, we moved on to create a full processing pipeline for the data set. Figure 3 shows an overview of the pipeline. Below, we discuss each of these pipeline steps in detail.

### 5.1 Eye-tracker

We first pre-processed the eye-tracking recordings by mapping gaze points onto the scene camera image. Here, we used *post-hoc* pupil detection. This allowed us to enhance accuracy by trimming any cases where the eye was obscured by dark eyelashes by adjusting the Region of Interest (ROI) to be closer to the edges of the eye. This was particularly useful for participants with dark, long eyelashes and with caregivers wearing mascara. We also used *post-hoc* pupil detection to ensure that the calibration for each session was accurate. Here, we used the Vis Circle plugin from the pupil player software to visualize gaze positions, mapping these positions onto the caregivers' world camera view. We could then verify that the gaze position overlapped the calibration marker, removing any mis-matching entries. If the number of calibration points dismissed by the software was over 40%, the data were excluded. After *post-hoc* calibration, we exported the following: (1) the play session video with gaze position overlaid on the video, (2) a .csv file consisting of the pupil and gaze coordinates and confidence, and (3) timestamps in NumPy format (for complete documentation, see Pupil Player documentation).

### 5.2 Annotation

We used the annotation plugin in the pupil player software to synchronize the parent and infant head camera videos. The movie clapperboard was used as a reference to annotate the synchronized onset of the play session for both parent and infants' head cameras. The offset was synchronized either at the end of the ten-minute play session or when the session ended (in case of infant fussiness). Any crying event for more than a minute was annotated to remove from the processing during the frame extraction phase (see below).

### 5.3 Frame extraction

The next step used the parent and infant raw .mp4 video, the corresponding timestamp files, the annotation file, and the gaze position file (for parents only). Synchronized frames were extracted from the videos in MATLAB using FFmpeg (frame extraction code available on GitHub). We compensated for any fluctuations in time by downsampling, using the caregiver or infant video as the referent signal, whichever had fewer frames. We also filtered any crying epoch that lasted more than a minute. Once the frames were synchronized, they were renamed with consecutive frame IDs. Following this, we generated a new gaze data file with normalized X and Y positions for each gaze point matching the frames from the parent video.

### 5.4 Machine learning

The synchronized video frames were evaluated by both MTCNN and YOLO on a high-performance computing cluster. MTCNN included two additional filtering steps to further improve the data quality:

1. When an item was detected for at least 3 frames (e.g., bounding box on parent's face), then detection ceased for a frame, and the item was detected again for 3 frames, the filtering step filled the missing frame. This reduced the number of false rejections (misses);
2. When the identification of items lasted for only a single frame, the filtering step discarded the detection. This served to minimize false positives.

### 5.5 Visualization and validation using BORIS

To verify MTCNN and YOLO performance, we manually processed data for four dyads from the UK and eight dyads from India. We visualized the MTCNN and YOLO predictions by converting the synchronized frames for each dyad into a video format at 90fps (to speed up manual coding). Each video consisted of the predictions visualized using green bounding boxes for toys and faces with the predicted accuracy (in %) and a blue gaze position with a blue extended bounding box around it. The gaze position for caregivers' eye-tracker was based on the gaze output from the pupil player. We created a gaze position for the infants' headcamera at the center of the frame because infants tend to fixate large, centrally-positioned objects. The gaze box was determined by the approximate central vision field of view (FOV), the FOV of the camera, and the camera sensor resolution. The camera FOV for pupil labs is 60 degrees and we assumed that the central human FOV is approximately 15 degrees. We, then, converted the raw gaze data obtained from pupil player processing and converted it to pixel coordinates for the central blue dot.

**Figure 4:**
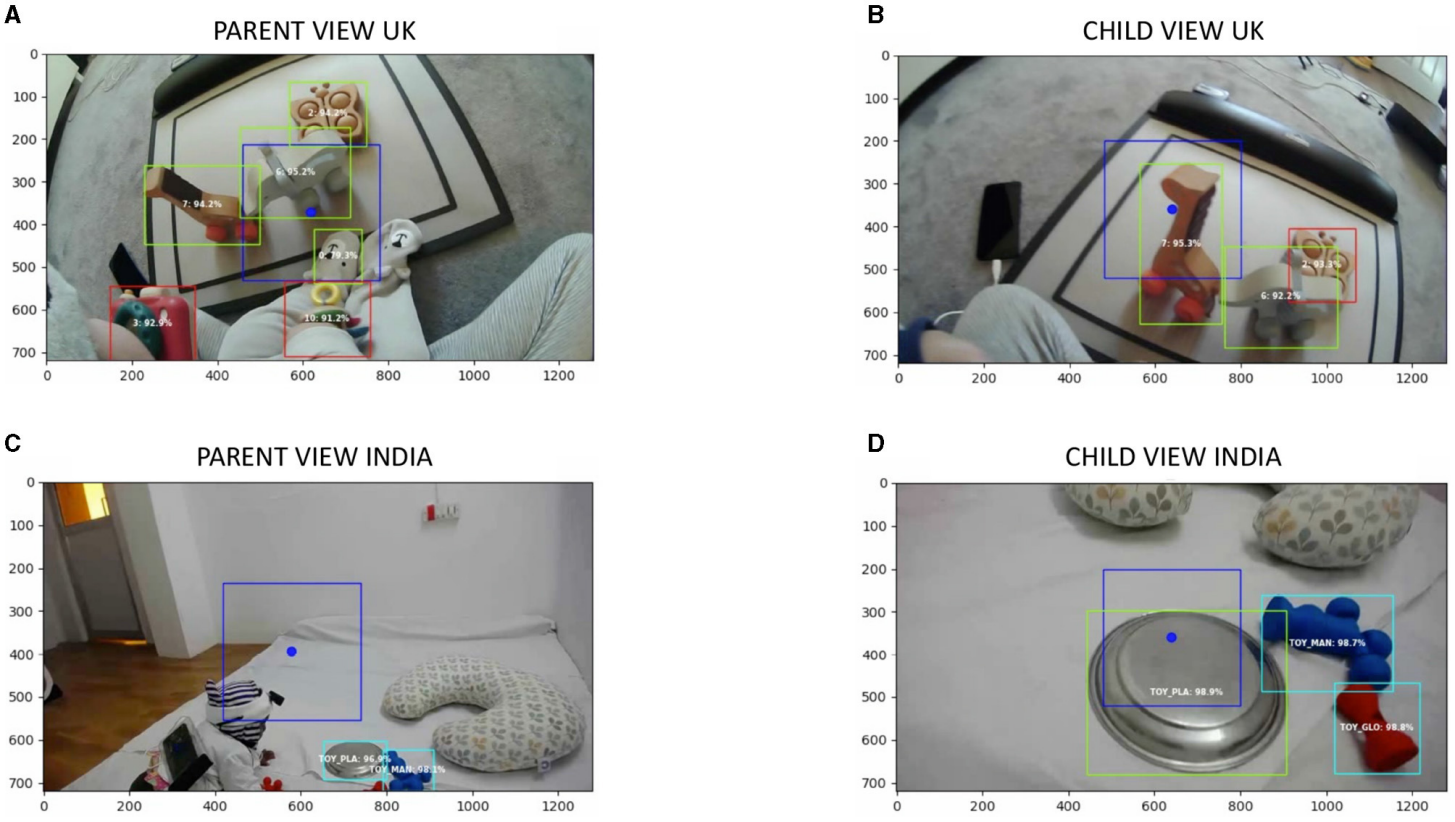
Example frames from machine learning visualization output for a dyad from the UK **(A, B)** and India **(C, D)**. **(A, B)** Depict a frame from the parent's and infant's view, respectively. The blue bounding box with a circle depicts the gaze. The red bounding boxes depict toys predicted by the algorithm that does not overlap with the parent/infant's gaze. Green bounding boxes depict toys and faces predicted by the algorithms that overlap the parent/infant's gaze. **(C, D)** Depict a frame from the parent's and infant's view, respectively from an Indian dyad. A blue bounding box with a circle depicts the gaze. The cyan bounding boxes depict toys predicted by the algorithm that does not overlap with the parent/infant's gaze. Green bounding boxes depict toys and faces predicted by the algorithms that overlap the parent's gaze. Each predicted bounding box consisted of the label for the object or face (in numbers for the UK and in letters for the India dyad) and well its corresponding prediction confidence in percentage.

Figure 4 shows example frames from a dyad from the UK (Figures 4A, B) and India (Figures 4C, D). Trained experimenters coded hits (correct detections), misses (incorrect rejection), false-positives, and true negatives for each video. For instance, in Figure 4A, detection of a face on the infants' onesize (label 0 with prediction confidence 79.3%) would be coded as a false positive, missed detection of toys puzzle and glow in image C would be labeled as a miss, and all other detections in all four images would be labeled as a hit. For, YOLO, each toy was coded twice, once from the parent's headcamera and once from the infant's headcamera. Results from this validation step are presented below.

### 5.6 Event detection

After validating our approach, we created event detection files for each prediction (MTCNN, YOLO) for each member of the dyad (parent, infant). The event detection script reads in the gaze report and predictions and creates a new .csv file that indicates which object each partner is looking at, outputting three columns: (1) the onset frame of each label, (2) the offset frame of that label, and (3) the corresponding label. An event was defined as a continuous series of 3 or more frames (or 99msec) looking at the same label (toy, face). In case there is more than one object in the dyad's view (e.g., Figures 4A, B), then the most central bounding box (within the blue gaze box) with the largest confidence was considered as the main object in view (e.g., toy elephant in Figure 4A and toy giraffe in Figure 4B).

### 5.7 Time is Very imPortant toolkit (TimeVP)

The next step in processing was to analyse the behavioral data to understand the dynamic interaction between parent and infant. Here, we used the Time is Very important (TimeVP) toolkit developed by the Developmental Intelligence Lab (Yu and Smith, 2016, see also) available on GitHub (https://github.com/devintel-lab/timevp). TimeVP uses the event files and computes key variables of interest. The toolbox provides visualization tools that can be crucial to checking data quality, validating pre-processing as well as examining patterns in the data (Yu et al., 2012). Next, it provides measures of visual cognition: (1) Mean Look Duration (MLD) at the target (toys, face), and (2) Switch Rate (SR) between targets (toys, face). Lastly, it enables the user to extract coupled behaviors to understand the temporal relations between two events such as episodes of joint attention led by parents vs. infants.

We ran three TimeVP scripts for each cohort (6 months UK, 6 months India, and 9 months India). The first scripts visualized the sequential temporal events. Next, we ran two individual scripts for parents and infants to compute individual and overall statistics such as proportion, duration and frequency of looks on a target. Note that we also used a final script in Study 2. This paired event script computed joint attention events and who led each joint attention episode.

## 6 Results

### 6.1 Evaluation of MTCNN accuracy

Results from manual coding for MTCNN are shown in Table 2. The mean accuracy across all dyads in the UK was 95.41% (SD = 0.04). The mean accuracy across dyads in India was 96.08% (SD = 0.04). Performance was good with both infants' head-cameras and with parents' head-cameras. Thus, MTCNN performed very well on our data sets.

**Table 2:** The proportion of false positives, misses, hits, and true negatives for MTCNN UK (top) and India (bottom).

**UK participant**	**False positive (%)**	**Miss (%)**	**Hit (%)**	**True negative (%)**	**Overall percent correct (%)**
Child1	1.6	1.3	6.4	90.7	**97.1**
Parent1	0.5	3.1	23.3	73.0	**96.3**
Child2	0.2	0.8	59.7	39.3	**99.0**
Parent2	0.4	8.6	54.8	36.1	**90.9**
Child3	0.7	6.6	1.9	90.8	**92.7**
Parent3	0.7	9.1	17.9	72.3	**90.2**
Child4	0.5	0.5	5.6	93.3	**98.9**
Parent4	1.3	0.6	25.3	72.9	**98.2**
**Mean**	**0.7**	**3.8**	**24.4**	**71.1**	**95.4**
**India participant**	**False positive (%)**	**Miss (%)**	**Hit (%)**	**True negative (%)**	**Overall percent correct (%)**
Child1	1.1	0.9	1.8	96.1	**97.9**
Parent1	1.9	9.8	15.3	73.0	**88.3**
Child2	1.0	5.7	1.8	89.4	**91.2**
Parent2	0.8	3.3	19.4	76.5	**95.9**
Child3	1.1	2.0	0.8	96.1	**96.9**
Parent3	0.7	5.4	20.3	73.7	**94.0**
Child4	1.4	0	4.2	94.4	**98.6**
Parent4	0.9	0.5	14.7	83.8	**98.5**
Child5	0.4	0.1	3.8	95.7	**99.5**
Parent5	0.8	1.3	23.6	74.4	**98.0**
Child6	1.4	0	0.2	98.4	**98.6**
Parent6	9.1	0.6	3.5	86.8	**90.3**
Child7	0.6	0	13.3	86.1	**99.4**
Parent7	1.7	1.0	20.6	76.7	**97.3**
Child8	2.3	0.9	4.9	91.9	**96.8**
Parent8	2.4	1.6	20.9	75.1	**96.0**
**Mean**	**1.7**	**2.1**	**10.6**	**85.5**	**96.1**

### 6.2 Evaluation of YOLO accuracy

Table 3 show results from manual coding for YOLO object recognition. The mean accuracy across all dyads in the UK was 92.61% (SD = 0.07), and the mean accuracy across dyads in India was 96.52% (SD = 0.06). YOLO performed well with both data from infants' head-cameras and parents' head-cameras.

**Table 3:** Proportion of false positives, misses, hits, and true negatives for toys from dyads in UK (top) and India (bottom).

**UK toys**	**False positive (%)**	**Miss (%)**	**Hit (%)**	**True negative (%)**	**Overall percent correct (%)**
TOY_BUT Child	0	3.1	61.5	0.35	**96.9**
TOY_BUT Parent	0	05.0	73.6	21.4	**95.0**
TOY_RAT Child	0.2	0.8	33.0	66.0	**99.0**
TOY_RAT Parent	0	4.7	29.1	66.2	**95.3**
TOY_CAM Child	0	11.3	24.3	64.4	**88.7**
TOY_CAM Parent	0	12.5	31.7	55.9	**87.6**
TOY_ELE Child	0	4.2	47.3	48.4	**95.7**
TOY_ELE Parent	0	8.1	17.5	74.4	**91.9**
TOY_GIR Child	0	10.0	43.7	46.3	**90**.0
TOY_GIR Parent	0	8.0	48.7	43.3	**92.0**
TOY_KET Child	0	2.7	79.9	17.4	**97.3**
TOY_KET Parent	0	18.3	75.3	6.4	**81.7**
TOY_APP Child	0	1.0	42.4	56.6	**99.0**
TOY_APP Parent	0	6.8	68.1	25.1	**93.2**
TOY_CUP Child	0	17.1	28.4	54.5	**82.9**
TOY_CUP Parent	0.1	27.4	32.1	40.4	**72.5**
TOY_TRA Child	0	2.5	65.8	31.7	**97**.5
TOY_TRA Parent	0.1	3.3	75.1	21.5	**96.6**
TOY_DUC Child	0	0.6	52.8	46.6	**99.4**
TOY_DUC Parent	0	0	69.6	30.4	**100.0**
**India toys**	**False positive (%)**	**Miss (%)**	**Hit (%)**	**True negative (%)**	**Overall percent correct (%)**
TOY_RAT Child	0	1.7	32.4	65.9	**98.3**
TOY_RAT Parent	0	9.1	69.7	21.3	**91.0**
TOY_PUZ Child	0	1.6	10.3	88.1	**98.4**
TOY_PUZ Parent	0	0.7	47.7	51.6	**99.3**
TOY_MAN Child	0	2.2	40.9	56.9	**97.8**
TOY_MAN Parent	0	0	43.8	56.2	**100.0**
TOY_PLA Child	0.5	1.1	36.3	62.1	**98.4**
TOY_PLA Parent	0	5.5	30.2	64.3	**94.5**
TOY_CAN Child	0.1	0	17.6	82.3	**99.9**
TOY_CAN Parent	0.1	4.6	38.8	56.5	**95.3**
TOY_SPO Child	0	3.9	5.6	90.5	**96.1**
TOY_SPO Parent	0	15.6	6.7	77.8	**84.5**
TOY_YEL Child	0	0.1	9.7	90.2	**99.9**
TOY_YEL Parent	0	6.9	49.4	43.8	**93.2**
TOY_GLO Child	0.3	3.5	8.6	87.6	**96.2**
TOY_GLO Parent	6.8	3.5	37.9	51.8	**89.7**

### 6.3 Quantifying the dyadic data using the TimeVP toolkit

As a first step in evaluating the quality of our data, we visualized the temporal data stream for parents and their infants during toy play (see Supplementary Figure 1). A few qualitative patterns were evident. Dyads with 6-month-old infants in the UK tended to look more toward their social partner's faces relative to 6-month-olds in India. There also seemed to be longer looking durations for the UK infants. For the 6-month-old dyads in the India cohort, there seemed to be fewer looks to social partners' faces relative to 9-month-olds in India.

**Figure 5:**
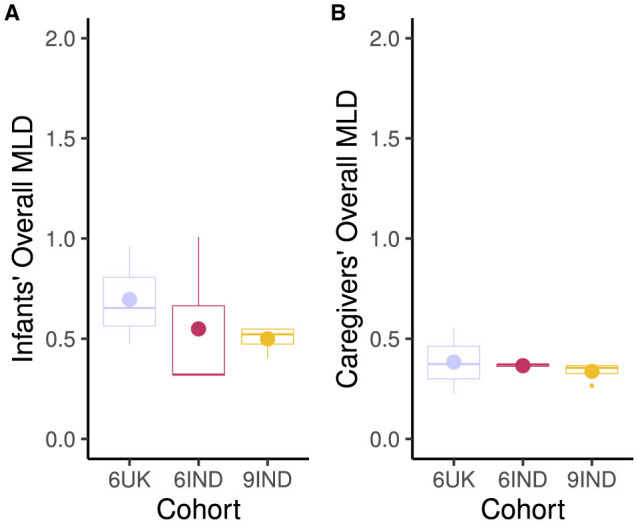
Mean Look Duration in seconds. **(A)** Infants' Mean Look Duration for all targets. **(B)** Caregivers' Mean Look Duration for all targets. Cohort 6UK represents 6-month-old infants from the UK; 6IND represents 6-month-old infants from India; 9IND represents 9-month-old infants in India. Dot in the boxplots represents the mean, and the horizontal line represents the median with lower and upper hinges showing the first and third quartiles, respectively.

Next, we looked at the overall Mean Look Duration for caregivers and infants across cohorts (6 months UK, 6 months India and 9 months India). Figure 5 indicates that 6-month-old infants from the UK had the longest MLD (M = 0.70, SD = 0.25). Within the Indian cohort, the 9-month-old infants (M = 0.50, SD = 0.07) had slightly longer MLD than the 6-month-old infants (M = 0.49, SD = 0.35) during the interaction. Caregivers' in the UK also had the longest MLD during the dyadic interaction (M = 0.38, SD = 0.16) compared to the other groups. This was followed by the caregivers of 6 month old infants (M = 0.36 SD = 0.01) and 9-month-old infants (M = 0.34, SD = 0.05) in India.

**Figure 6:**
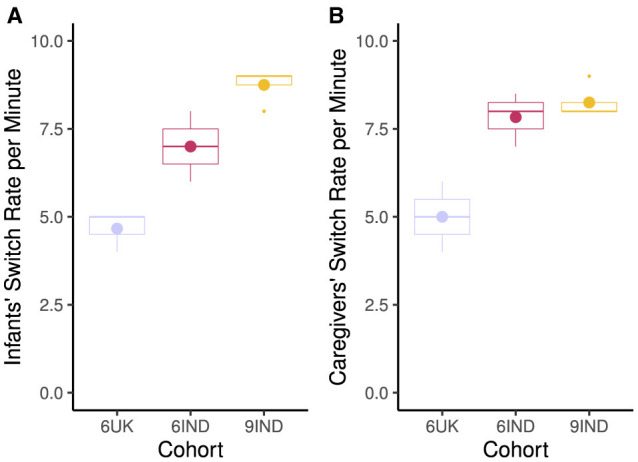
Switch rate between the target (toys, faces) per minute. **(A)** Infants switch rates per minute. **(B)** Caregivers switch rate per minute. Cohort 6UK represents infants and caregivers of 6-month-old infants from the UK; 6IND represents infants and caregivers of 6-month-old infants from India; 9IND represents infants and caregivers of 9-month-old infants in India. Boxplot details are same as in Figure 5.

In terms of switch rate, Figure 6 shows that the highest switch rate per minute was for the 9-month-old infants (M= 8.75; SD = 0.50) and their caregivers (M = 8.25; SR = 0.50) from India. 6-month-old infants (M = 7.0; SD = 1.83) and their caregivers (M = 8.0; SD = 0.82) had lower switch rates with 6-month-old UK dyads showing the smallest number of switches per minute (M = 4.67; SD = 0.58, for infants and M = 5.0; SD = 1.0, for caregivers).

## 7 Discussion

Study 1 aimed to create a single pipeline to process real-world, dynamic, caregiver-infant interactions recorded using head-mounted eye-trackers in both a high- and a low-resource setting. In this process, we provided solutions to two key issues in developmental science: (1) diversifying our participant pool by going beyond a western, middle-class society and (2) processing parent-infant interaction in a less laborious and more efficient way that can be used on large data sets including long-duration videos.

The pipeline we developed processed the dynamic interaction between caregivers and their infants in a non-biased way that was transferable across cultural settings. We, first, demonstrated that parent-infant interaction videos from a first-person perspective can be obtained from both high (urban UK) and low (rural India) resource settings. Next, we generated gaze reports for caregivers' eye-trackers and synchronized caregiver-infants interaction videos. Once the synchronized frames were extracted, we used machine learning algorithms to detect faces and recognize toys in the videos.

Note, we focused on face detection and not face recognition as there is only one other face (either infants or their caregivers) in the videos. Additionally, we used pre-trained face detection models (MTCNN) that have already been labeled on 32,203 images with 393,703 faces labeled, making the process of face detection less laborious. However, using a pre-trained model does not come without limitations.The initial accuracy (pre-filtering stage) for our UK and India cohort was 70% and 60% respectively. While adding the filtering step increased the accuracy by 20%–28%, we suspect that the WIDER face dataset used by MTCNN to create a pre-trained model consists of fewer infant faces (i.e., mostly adult and child faces). It would be useful for future work to explore this by running two separate models of equal quality on caregiver and infant faces and comparing the accuracy. Alternatively, the MTCNN model can be updated by training infant faces (across ethnicity) and adding the weights to the existing pre-trained model.

Next, we showed that a YOLO object recognition algorithm performed remarkably well across cohorts. We acknowledge that the pipeline is not completely automated and requires the user to make decisions, particularly at the filtering step, after making a qualitative pass on the output obtained from the machine learning algorithm. We expect, however, that the filtering steps applied here will be applicable to other projects. Moreover, given that training the network can be time-consuming as it requires labeling and annotating a large dataset, setting up a larger database could solve this issue. Training up a network with a large set of commercially available standardized toys worldwide could enable the same network to generalize across multiple studies. This would enable researchers across borders to simply use a selection from the standardized toy set and run the pipeline without any additional training.

Future research could also expand the pipeline by looking at other features such as detecting hands (Zhang et al., 2020a) or facial expression of emotions. For instance, work by Yu and Smith (2013) in western settings has noted that the infants' and toddlers' visual field often consists of hands and hands manipulating toys. Similarly, a study by Jayaraman et al. (2017) noted an age-related increase in the input of hands in infants' visual view. Thus, adding hand detection would enable us to possibly replicate these findings in a low-resource setting. Such work would also enable researchers to understand how deaf infants and their caregivers use sign language (Brooks et al., 2020).

In terms of data analyses, the TimeVP toolbox successfully enabled us to compute measures of interest such as MLD and switch rate. Given that the aim of this study was to construct a methodological pipeline yielding key measures of interest, we chose to report only the descriptive statistics from a small number of participants. Interestingly, however, this small sample revealed several similarities and differences across cohorts. For instance, infants in the UK tend to show longer MLD to targets with lower switch rates. Mean look durations and switch rates are typically negatively correlated in western samples, so this is a good validation check on the data set (Rose et al., 2002; Yu and Smith, 2017). To further evaluate the utility of this pipeline, Study 2 applied the pipeline to a large data set (sample size >20) across the two resource settings. This allowed us to statistically examine the similarities and differences across the three cohorts.

## 8 Study 2: applying the dyadic visual cognition pipeline across cultures

The goal of the present study was to explore and understand the similarities and differences between measures of dyadic visual cognition across cultures. This is important as key visual cognitive measures such as joint attention havebeen conceptualized and operationalised in different manners across studies, time and socio-cultural contexts (Siposova and Carpenter, 2019; Bard et al., 2021). While there is a general consensus that the infant's ability to engage in joint attention lays the groundwork for developmental advances such as language learning (Carpenter et al., 1998; Mundy and Gomes, 1998), social cognition (Mundy and Newell, 2007), and theory of mind (Nelson et al., 2008), characteristics of joint attention in social interaction are complex and can emerge via many pathways. These include initiating and responding to joint attention, maintaining attention to the common target of interest, ending the joint attention bout as well as shifting to or disengaging from an object. These instances of coordinated joint attention, in turn, predict infants' engagement in sustained attention (Yu and Smith, 2016) and vocabulary development (Abney et al., 2017). For instance, a study by Yu and Smith (2016) explored the influence of social context on parent-infant interaction during a free-flowing play session. They found that when the parent visually attended to the same object to which the infant was attending, infants attended to that object for longer than in the case when the parent was attending to a different object. Caregivers' labeling an object in such instances also predicted infants' later vocabulary.

The current study examines dyadic visual cognition in a large sample of infants in high- and low-resource settings. This comparison is important as most of our knowledge about joint attention comes from research conducted with infants from what Henrich et al. (2010) described as the WEIRD (i.e., Western, Educated, Industrialized, Rich, and Democratic) setting (Bard et al., 2021, also see). Here, we use the objective measures of visual cognition extracted using the machine learning pipeline developed in Study 1 and examined them through the lens of the eco-cultural model of parenting (Keller et al., 2005; Keller, 2007).

In the eco-cultural model of parenting, Keller (2007) identified two key parenting styles, distal and proximal. Distal parenting style involves exclusive focus on face-to-face interaction, object simulation, and child-centered responsiveness as well as emphasizing “positive” affect during caregiver-infant interaction. This parenting style has typically been characteristic of the urban, middle-class families from western cultures. The approach entails a pedagogical way of playing with the child, that is, with the aim of teaching the child (Lancy, 2010). On the other hand, the proximal parenting style uses modalities such as body contact, tactile simulation, focus on calming and soothing the infant as well as a directive, adult-centered interaction. In their extensive work on understanding caregiver-infant interaction in different social contexts, this has been typically characteristic of rural, subsistence farming families of traditional villages (e.g., work in rural Gujarat in India Abels et al., 2017). Proximal parenting style takes on the approach of responding to their infant's distress signals, almost always by breast-feeding or doing something to calm down the infant, that is, “*a quiet baby is a healthy baby”* (Lancy, 2007, p. 275).

Other research examining the cultural similarities and differences in maternal parenting have also looked at parenting style and conversational patterns between mothers and their 3-month-old infants in Delhi and Berlin using cultural models of autonomy and relatedness. This work found that mothers in Delhi shaped the interactions with their 3-month-old infants by leading and defining the structure of the play (Keller et al., 2010). By contrast, infants in Berlin tended to take on an active role in leading the interaction by directing their mothers' attention to, for instance, a toy. The autonomous model, an extension of the distal parenting style, involves caregivers addressing their infants as someone with agency and emphasizing the development of autonomy, wherein, infants actively direct and initiate interactions. On the other hand, parenting style in urban Delhi consisted of a combination of autonomous-relatedness, that is, mothers having higher formal education (linked to the autonomous style) as well as a bias toward traditional family ties and kinship (linked to relatedness). This was further indicated in the maternal parenting style. That is, while the mothers' showed a bias for proximal caregiving style (i.e., more body contact), they did not significantly differ in the use of both proximal and distal parenting style during play. Thus, they used body contact, tactical stimulation as well as playing with the object. By contrast, mothers in Berlin showed a clear bias toward distal (and autonomous) parenting style.

Given these differences in social interaction across cultural settings, we expected to find differences in our measures of interest across cultures. It is important to note, however, that the goal of this study is not to make inferences regarding which interaction style is better or worse. Instead, we focus on evaluating the similarities as well the differences in how parents and infants deploy their visual attention during instances of social interactive play. In line with the work by Yu and Smith (2016), we use the term “joint attention” to refer to a process in which caregiver and their infants focus their visual attention, together, on a common object or each others' faces at the same time (also see Yu and Smith, 2013).

## 9 Method

### 9.1 Participants

For the UK sample, we present data from 25 dyads of 6-month-old infants (15 females) recruited by the Developmental Dynamics Lab at the University of East Anglia in the UK through the same procedure as in Study 1. Parents were informed of the experiment's aim and procedure, and written consent was obtained. Remuneration comprised of 20 pounds, travel expenses, a t-shirt and a toy for each participant.

For the Indian sample, we report data from 32 3-month-old infants ±15 days (17 females) and 37 9-month-old ±15 days (15 females) infants and their caregivers. Characteristics of the sample from both India and the UK are summarized in Table 4. The procedure and protocol for the recruitment of participants were the same as in Study 1.

**Table 4:** Summary of key demographic features of our participants from UK and India in percentage.

	**6UK**		**6IND**	**9IND**
**N**	**25 (15 females)**	**N**	**31 (17 females)**	**34 (15 females)**
**Mother education (%)**		**Mother education (%)**		
Left school	4	Primary pass	48.4	47.1
Up to A levels	16	Secondary and higher education	51.6	52.9
Bachelor's degree	36	**Father education (%)**		
Masters degree	28	Primary pass	48.4	47.1
Doctorate or professional degree	16	Secondary and higher pass	51.6	52.9
**Father education (%)**		**Caste (%)**		
Left school	0	General	6.5	2.9
Up to A levels	24	Other backward class (OBC)	29	41.2
Bachelor's degree	44	Schedule caste/ tribe (SC/ST)	64.5	55.9
Masters degree	20	**Family income in INR (%)**		
Doctorate or professional degree	3	< 100,000	77.4	73.5
**Ethnic group (%)**		>= 100,000	22.6	26.5
African	0			
Asian	4			
Mixed	8			
White	88			
**Family income median in GBP (%)**				
< 20,000	12			
>= 20,000 & < 40,000	20			
< = 40,000	68			

6UK denotes dyads with 6-month-old infants from the UK, 6IND denotes dyads with 6-month-old infants from India and 9IND denotes dyads with 9-month-old infants from India.

### 9.2 Materials and procedure

The materials and procedure for capturing caregiver-infant interactions were the same as in Study 1.

### 9.3 Data processing

We processed the data through the pipeline developed in Study 1. The event files were then processed using the TimeVP toolbox (Yu and Smith, 2016, also see). The data were visualized as an initial data quality check (see Supplementary Figure 2). Here we discovered that there were more gaps in the 6UK time series (i.e., more white space in the figure reflecting looking to non-targets). This may reflect differences in the context of the interactions. Recall that UK dyads were tested in the home, while Indian dyads were tested in a lab with few objects in the surroundings. Thus, it is likely that UK infants and caregivers looked at other objects in their surroundings more often—objects which were not captured by the machine learning approach. In the next analysis step, we computed measures of Mean Look Duration (MLD), Switch Rate (SR) and episodes of caregiver-led and infant-led joint attention.

### 9.4 Analytic strategy

The analyses are divided into three sections. In the first section, we look at scores of the overall mean look duration and switch rate per minute, including a focus on both faces and toys for both caregivers and their infants across the three cohorts (6 UK, 6 IND, 9 IND). In Section 2, we shift the focus to exploring episodes of joint attention, addressing differences between the three groups in terms of the total number of episodes of joint attention, the proportion of the total in which the joint attention was led by the infants, and the proportion which were terminated by the infants. Across the first two sections, we performed Welch two-sample *t-*tests to compare the means across cohorts. We use Welch's t-test because it is more robust than Students' *t*-test when sample sizes are unequal and does not rely on the assumption of equal variance between groups (Delacre et al., 2017). Additionally, effect sizes are calculated using Cohen's *d* (Sullivan and Feinn, 2012).

In Section 3, we analyse differences in infants' mean look duration toward toys and faces across three conditions: when the infant was looking at toys or caregivers' faces by themselves (i.e., without the caregiver looking at the same target), when the infant initiated the joint attention episode, and when the caregiver initiated the joint attention episode. The same analyses on mean look duration toward toys and faces were then repeated for the caregivers. As with natural behavior, the mean-looking duration for participants was such that most were very brief and some very long. Therefore, to compare the MLD between cohorts, we used the Mann-Whitney *U*-test due to the skewed distribution of looking behavior rather than Welch's two-sample *t*-tests that assume normality (Gibbons and Chakraborti, 2011; Yuan et al., 2019). Here, the effect size was determined using the Rank-Biseral Correlation (Kerby, 2014).

## 10 Results

### 10.1 Overall mean look duration and switch rate

**Figure 7:**
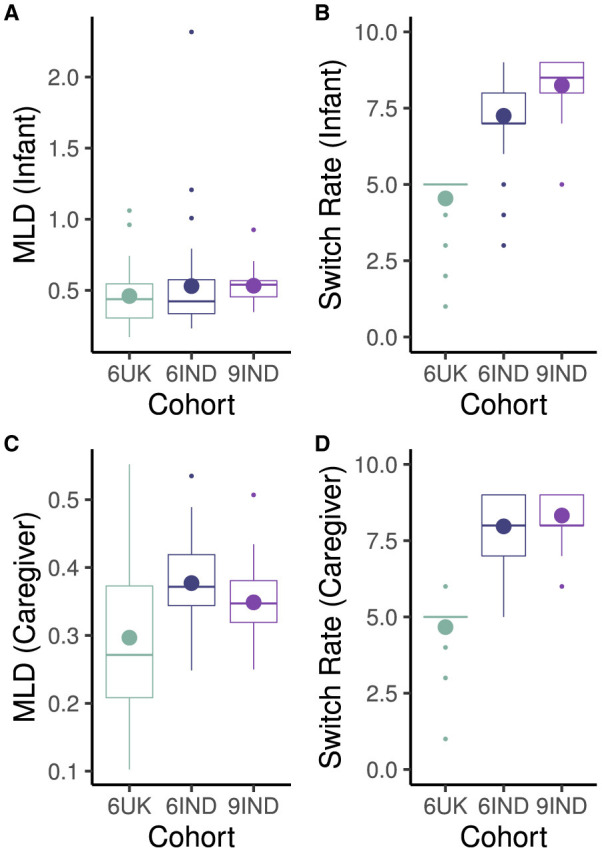
Overall Mean Look Duration in seconds and Switch Rate per minute across cohort for infants **(A, B)** and caregivers **(C, D)**. Cohort 6UK represents caregivers of 6-month-old infants from the UK; 6IND represents caregivers of 6-month-old infants from India; 9IND represents caregivers of 9-month-old infants in India. Dot in boxplots represent mean and horizontal line represents represent median with the lower and upper hinges showing first and third quartiles, respectively.

Scores of the overall mean look duration and switch rate per minute for infants and caregivers across the three cohorts are shown in Figures 7A, B. Welch two sample t-tests on the the MLD of infants across the three cohorts found no significant differences (*t*_6IND, 9IND_(42.79) = –1.38, *p* = 0.18, *d* = –0.35, 95% CI [-0.84, 0.13]; *t*_9IND, 6UK_ (30.53) = 1.46, *p* = 0.15, *d* = –0.44, 95% CI [–0.96, 0.09]; *t*_6IND, 6UK_ (49.08) = 0.20, *p* = 0.84, *d* = 0.05, 95% CI [–0.48, 0.59]). On the other hand, the switch rate between targets (e.g., between toys, or between toys and faces) differed significantly across cohorts. Infants in the 9-month-old cohort from India had higher switch rate compared to the 6-month old infants in India (*t*_6IND, 9IND_(54.09) = –2.94, *p* < 0.01, *d* = –0.74, 95% CI [-1.23, –0.24]). In turn, the 6-month-old infants from India displayed a significantly higher switch rate compared to those from the UK(*t*_6IND, 6UK_(52.98) = 8.46, *p* < 0.001, *d* = 2.22, 95% CI [1.53, 2.89]). Note that the low switch rate for the 6UK infants is consistent with the observation that the UK cohort had more periods of looking to non-targets. Looks to non-targets would create longer gaps between events, thereby lowering the shift rate per minute.

Analyses for caregivers' MLD revealed that the caregivers' of 6-month-old infants in India tended to have longer MLD than caregivers of 6-month-old infants in the UK (*t*_6UK, 6IND_(34.74) = 2.85, *p* < 0.01, *d* = 0.83, 95% CI [0.27, 1.37]). However, there were no differences between the two Indian cohorts (*t*_6IND, 9IND_(56.94) = 1.82, *p* = 0.074, *d* = 0.45, 95% CI [–0.03, 0.93]; also see Figure 7C) nor between the MLD of caregivers from the 6-month cohort in the UK and 9-month old cohort in India (*t*_6UK, 9IND_(28.88) = –1.95, *p* = 0.061, *d* = –0.59, 95% CI [–1.12, –0.07]).

In term of switch rate, caregivers of 9-month old infants in India switched at a significantly higher rate than caregivers in the UK (*t*_6UK, 9IND_(42.04) = –15.56, *p* < 0.001, *d* = –4.26, 95% CI [–5.18, –3.33]; see Figure 7D). Caregivers in the 6-month-old infant cohort also differed in their switch rate (*t*_6UK, 6IND_(50.65) = 12.49, *p* < 0.001, *d* = 3.36, 95% CI [2.52, 4.17]) such that caregivers of the Indian cohort showed significantly higher switch rate. However, there was no difference between the two cohorts in India (*t*_6IND, 9IND_(58.49) = –1.63, *p* = 0.11, *d* = –0.40, 95% CI [–0.88, 0.08]). Again, this is consistent with the observation of greater white space in the raw data visualization in the UK cohort which would lead to lower switch rates in the UK caregivers.

### 10.2 Proportion of joint attention episodes

**Figure 8:**
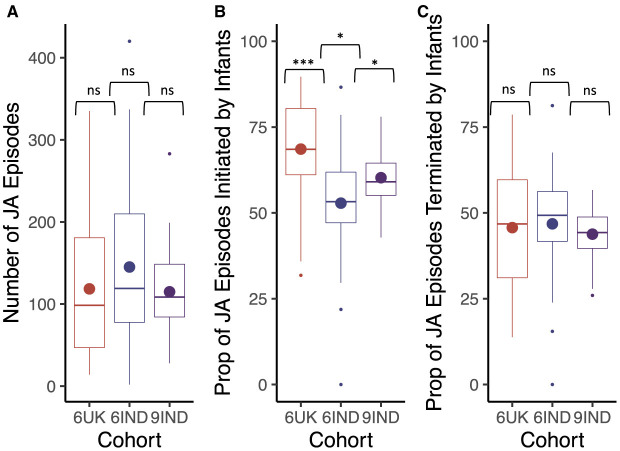
Box plots indicate **(A)** the total number of joint attention episodes in each cohort, **(B)** the proportion of joint attention episodes imitated by infants in each cohort (in percentage) and **(C)** the proportion of joint attention episodes terminated by infants in each cohort (in percentage). Cohort 6UK represents caregivers of 6-month-old infants from the UK; 6IND represents caregivers of 6-month-old infants from India; 9IND represents caregivers of 9-month-old infants in India. Boxplot details are same as in Figure 7. Ns indicates *p* > 0.05, ^*^indicates *p* < 0.05, ^**^indicates *p* < 0.01, ^***^indicates *p* < 0.001.

Results revealed no significant differences between the cohorts in regards to the overall number of joint attention episodes (*t*_6IND, 9IND_(50.05) = 1.84, *p* = 0.07, *d* = 0.50, 95% CI [–0.05, 1.05]; *t*_9IND, 6UK_ (33.91) = 1.91, *p* = 0.07, *d* = 0.59, 95% CI [–0.02, 1.20]; *t*_6IND, 6UK_ (29.89)= 0.50, *p* = .62, *d* = 0.16, 95% CI [–0.44, 0.76]; Figure 8A). However, the proportion of infant-led joint attention episodes differed significantly across the three cohorts, with 6-months-old infants from India having a significantly smaller proportion of infant-led joint attention episodes than their 9-month-old counterparts (*t*_6IND, 9IND_(40.99) = –2.24, *p* < 0.05, *d* = –0.57, 95% CI [–1.05, –0.08]). In turn, 9-month-old infants from India led a significantly smaller proportion of joint attention episodes compared to the UK cohort (*t*_6UK, 9IND_(30.70) = 2.53, *p* < 0.05, *d* = 0.75, 95% CI [0.22, 1.29]; also see Figure 8B). Lastly, the proportion of joint attention bouts terminated by infants did not differ by cohort (*t*_6UK, 6IND_(44.84) = –0.23, *p* = 0.82, *d* = –0.06, 95% CI [–0.59, 0.47]; *t*_6IND, 9IND_(44.41) = 0.97, *p* = 0.34, *d* = 0.25, 95% CI [–0.23, 0.72]; *t*_6UK, 9IND_(28.60) = 0.47, *p* = 0.64, *d* = 0.14, 95% CI [–0.37, 0.66]; Figure 8C ).

### 10.3 Infants' mean look durations for faces and toys across contexts

**Figure 9:**
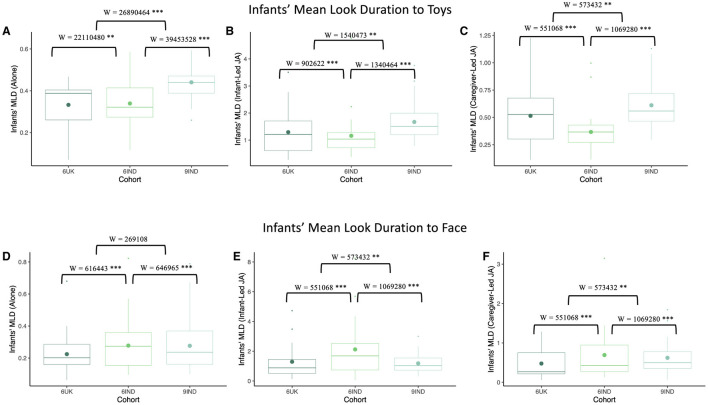
Comparing infants MLD using Mann-Whitney-U test across the three cohorts. The top row depicts infants' Mean Look Duration (MLD) to toys when **(A)** looking alone **(B)** looking with a caregiver in an infant-led joint attention episode **(C)** looking with a caregiver in a caregiver-led joint attention episode. The bottom row depicts infants' Mean look Duration to caregivers' faces when **(D)** looking alone **(E)** looking at each others' faces in infant-led joint attention episodes **(F)** looking at each others' faces in caregiver-led joint attention episodes. Boxplot details are same as in Figure 7. Blank indicates *p* > 0.05, ^*^indicates *p* < 0.05, ^**^indicates *p* < 0.01, ^***^indicates *p* < 0.001.

Mann-Whitney-U test was carried out for assessing differences between the groups and effect size were calculated using rank-biseral correlation to indicate the strength of the effect (CI 95%). As shown in Figure 9A, infants' MLD when looking to toys differed significantly across cohorts in the “looking alone” context (i.e., when visually exploring objects outside of a joint attention bout). In particular, 9-month-old infants from India showed the longest MLD directed at toys, and 6-month-old infants from India showed the shortest MLD when looking at toys, with the UK sample falling between both. Differences between all groups were significant (W_6UK, 9IND_ = 26,890,464, *p* < 0.001, *r* = –0.11, CI [–0.13, –0.09]; W_6IND, 9IND_ = 39,453,528, *p* < 0.001, *r* = –0.14, CI [–0.16, –0.12]; W_6UK, 6IND_ = 22,110,480, *p* < 0.001, *r* = 0.03, CI [0.01, 0.05]). When infants initiated the joint attention episodes, 9-month-old infants in India once again showed significantly longer MLD to the toys compared to 6-month-old infants in both India and the UK (W_6UK, 9IND_ = 1,540,473, *p* < 0.001, *r* = –0.05, CI [–0.09, –0.01]; W_6IND, 9IND_ = 1,340,464, *p* < 0.001, *r* = –0.19, CI [–0.23, –0.15]) . Among the 6-month-old infants, those from India showed significantly shorter MLD than their UK counterparts (W_6UK, 6IND_ = 902,622, *p* < 0.001, *r* = –0.13, CI [–0.17, –0.09]; see Figure 9B). Finally, in caregiver-led joint attention episodes, as shown in Figure 9C, the 9-month-old infants once again displayed longer MLD than the two 6-month-old cohorts (W_6UK, 9IND_ = 573,432, *p* < 0.001, *r* = –0.07, CI [–0.12, –0.02]; W_6IND, 9IND_ = 1,069,280, *p* < 0.001, *r* = –0.18, CI [–0.22, –0.14]). Moreover, the 6-month-old infants in India displayed shorter MLDs than the UK group (W_6UK, 6IND_ = 551,068, *p* < 0.001, *r* = –0.11, CI [–0.15, –0.06]). Thus, across all three contexts we found similar patterns. Note, however, that MLDs overall were shortest in the “looking alone” context, longer during caregiver-led joint attention episodes, and longer still during infant-led joint attention episodes (see scale changes along the y-axes in Figure 9).

When looking at the caregiver's faces in the “looking alone” context, the 6-month-old infants from India showed significantly longer MLD compared to the 6-month-old infants in the UK (W_6UK, 6IND_ = 616,443, *p* < 0.001, *r* = 0.09, CI [0.04, 0.14]) and the 9-month-old infants in India (W_6IND, 9IND_ = 646,965, *p* < 0.001, *r* = 0.13, CI [0.08, 0.18]). Results indicated no significant difference in the MLD between the latter two cohorts (W_6UK, 9IND_ = 269,108, *p* = 0.112, *r* = 0.05, CI [–0.01, 0.11]; see Figure 9D). As shown in Figure 9E, during episodes of joint attention initiated by the infants, 6-month-old infants from India spent a significantly longer time looking at their caregiver's faces compared to the two other groups (W_6IND, 9IND_ = 182,134, *p* < 0.001, *r* = 0.35, CI [0.28, 0.42]; W_6IND, 6UK_ = 349,597, *p* < 0.001, *r* = 0.19, CI [0.13, 0.25]). Moreover, 6-month-old infants from the UK had significantly longer MLDs focused on their caregivers' faces compared to the 9-month Indian infants (W_6UK, 9IND_ = 79,600, *p* < 0.001, *r* = 0.12, CI [0.04, 0.21]). Finally, as in the other contexts, when caregivers led the joint attention bouts, 6-month-old infants from India tended to look for longer at their caregivers' faces than infants in the other two cohorts ((W_6IND, 9IND_ = 1,069,280, *p* < 0.001, *r* = –0.18, CI [–0.22, –0.14]; W_6IND, 6UK_ = 551,068, *p* < 0.001, *r* = –0.11, CI [–0.15, –0.06]; see Figure 9F). In contrast to the analysis of infant-led joint attention, the MLD of 6-month-old infants from the UK was significantly shorter than that of 9-month-old infants in India (W_6UK, 9IND_ = 573432, *p* < .001, *r* = -0.07, CI [-0.12, -0.02]). Once again, we note that although some of these patterns were consistent across contexts (e.g., 6-month-old infants from India showed longer looking to faces than the other groups), the MLD varied across contexts in a manner similar to looking at toys with the shortest looking in the “looking alone” context, longer looking during caregiver-led joint attention episodes, and the longest looking during infant-led joint attention episodes.

### 10.4 Caregivers mean look durations for faces and toys across contexts

**Figure 10:**
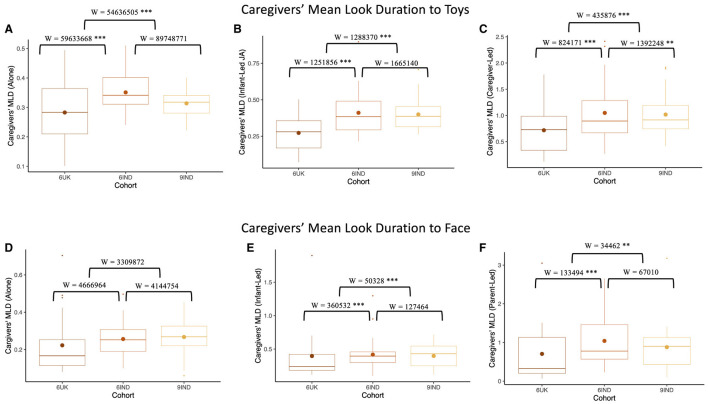
Comparing caregivers' MLD using Mann-Whitney-U test across the three cohorts. The top row depicts Caregivers' Mean Look Duration (MLD) to toys when **(A)** looking alone, **(B)** looking with caregiver in infant-led joint attention episode, and **(C)** looking with caregiver in caregiver lead joint attention episode. The bottom row depicts infants' Mean look Duration to caregivers' faces when, **(D)** looking alone, **(E)** looking at each others' faces in infant-led joint attention episodes, and **(F)** looking at each others' faces in caregiver-led joint attention episodes. Boxplot details are same as in Figure 7. Blank indicates *p* >0.05, ^*^indicates *p* < 0.05, ^**^indicates *p* < 0.01, ^***^indicates *p* < 0.001.

As with infants' looking, we examined how caregivers' look durations varied across contexts when looking at toys and faces. Results showed that caregivers' of 6-month-old infants in the UK had significantly shorter MLDs to toys in the “looking alone” context, compared to caregivers of Indian cohorts (W_6UK, 9IND_ = 54,636,505, *p* < 0.001, *r* = –0.14, CI [–0.15, –0.12]; W_6UK, 6IND_ = 59,633,668, *p* < 0.001, *r* = 0.13, CI [0.11, 0.14]; see Figure 10A). However, no significant difference in MLD to toys was found between the two Indian cohorts (W_6IND, 9IND_ = 89,748,771, *p* = 0.724, *r* = –0.002, CI [–0.02, –0.01]). With regards to caregivers' MLD to toys in joint attention episodes led by infants, as shown in Figure 10B, caregivers in the UK cohort once again displayed significantly shorter MLD than those in both Indian cohorts (W_6UK, 9IND_ = 1,288,370, *p* < 0.001, *r* = –0.21, CI [–0.24, –0.17]; W_6UK, 6IND_ = 1,251,856, *p* < 0.001, *r* = 0.21, CI [0.17, 0.25]). There were also no significant difference in the MLD of caregivers between the two Indian cohorts (W_6IND, 9IND_ = 1,665,140, *p* = 0.741, *r* = 6,400, CI [–0.03, 0.04]). Finally, in the case of caregiver-led joint attention episodes, the MLD of caregivers' from the UK was significantly shorter than the MLD of 6- and 9-month caregivers in India (W_6UK, 9IND_ = 435,876, *p* < 0.001, *r* = -0.21, CI [–0.24, –0.17]; W_6UK, 6IND_ = 824,171, *p* < 0.001, *r* = 0.34, CI [0.29, 0.38]). However, unlike in the previous results, caregivers of 6-month-old infants in India displayed longer MLD than those in the 9-month cohort (W_6IND, 9IND_ = 1,392,248, *p* < 0.001, *r* = 0.06, CI [0.02, 0.10]; see Figure 10C). As with infants' looking to toys, we found variation in looking across contexts; here, however, look durations were longest in the case of caregiver-led joint attention episodes.

No significant differences were found on caregivers' MLD toward their infants' faces in the “looking alone” context (W_6UK, 9IND_ = 3,309,872, *p* = 0.556, *r* = 9,500, CI [–0.02, 0.04]; W_6IND, 9IND_ = 4,144,754, *p* = 0.329, *r* = 0.02, CI [–0.02, 0.05]; W_6UK, 6IND_ = 4,666,964, *p* = 0.706, *r* = 5590, CI [–0.02, 0.03]; see Figure 10D). Within joint attention episodes initiated by infants, caregivers of the UK group showed significantly shorter MLD to their infants' faces compared to caregivers in the two Indian groups(W_6UK, 9IND_ = 50,328, *p* < 0.001, *r* = –0.29, CI [–0.37, –0.21]; W_6UK, 6IND_ = 360,532, *p* < 0.001, *r* = 0.23, CI [0.17, 0.28]). Caregiver MLD to their infants' faces did not differ between the two Indian cohorts (W_6IND, 9IND_ = 127464, *p* = 0.193, *r* = –0.05, CI [–0.13, 0.03]; see Figure 10E). As shown in Figure 10F, during caregiver-led joint attention, caregivers' from the UK displayed shorter MLD toward their infants' faces compared to the Indian caregivers (W_6UK, 9IND_ = 34,462, *p* < 0.001, *r* = –0.16, CI [–0.25, –0.06]; W_6UK, 6IND_ = 133,494, *p* < 0.001, *r* = 0.16, CI [0.09, 0.23]). Once again, caregivers from the two Indian cohorts did not significantly differ (W_6IND, 9IND_ = 67,010, *p* = 0.654, *r* = 0.02, CI [–0.07, 0.11]). We note that, once again, caregiver MLDs were longest in the caregiver-led joint attentional context.

## 11 Discussion

Study 2 yielded a rich array of findings using the pipeline developed in Study 1. These findings help us to (1) validate the visual dyadic pipeline with a larger data set and (2) explore how infants and their caregivers deploy visual attention during dyadic play across cultures (urban UK and rural India) and cohorts (6-month-old infants, 9-month old infants). Overall, findings indicated consistent trends in the deployment of visual attention by caregivers and their infants that reveal a complex interplay of influences on caregiver-infant interaction.

In regards to overall MLD which quantifies sustained looking toward the target of fixation, we found no overall differences for infants across groups suggesting that they deployed their visual attention similarly. At face value, this is remarkable given the many differences in cultural context that exist between the urban UK and rural India settings. On the other hand, analyses of caregiver MLD showed no differences between the two Indian cohorts but shorter MLDs in the UK compared to the 6-month-old India cohort. In the visual cognition literature, MLD has been interpreted in at least two ways: as indicative of the duration of sustained attention bouts (e.g., Yu and Smith, 2016) or as an index of speed of visual processing (e.g., Rose et al., 2002). In this context, UK caregivers may either have shorter bouts of sustained attention or they engage in faster visual processing. Few studies have examined the role of caregivers' visual processing and how this might shape infant-caregiver interactions. Thus, this is an important area requiring a more detailed look at adult visual cognitive processing.

When looking at the overall switch rate, caregivers and infants displayed a similar pattern where the Indian cohorts switched more between targets of fixation than the UK cohort. These apparent cultural differences in the deployment of visual attention need to be interpreted with caution, however. Typically, MLD and switch rate are negatively correlated such that longer MLD is related to fewer switches (or slower disengagement) (Colombo et al., 1991). In contrast, we found that caregivers with higher MLD also tended to have a higher switch rate. A key point to consider here is that the play sessions occurred in different contexts in India vs. the UK. Both Indian groups completed the play session in the same controlled environment, while British families carried out the study in their own homes. This matters because the measure of switch rate only takes into account switches between targets defined a priori in the TimeVP toolbox. Thus, if participants switched between targets of interest, the switch was computed. However, if participants shifted from a target of interest to an undefined target, no shift was recorded. Given that the rooms in which British families completed the study had many more objects (e.g., TV, sofa, photographs) which were not included in TimeVP as targets of interest, multiple instances of switches of attention are likely to have been missed.

When looking at differences between switch rates for the Indian groups, older infants displayed higher switch rates but no difference was found for caregivers. The higher switch rates for 9-month-old infants in India suggests that there are changes in looking dynamics as infants develop. For instance, studies with Western samples show that older infants typically show a faster rate of switching between objects (Rose et al., 2001, 2002). There are also likely to be improvements in motor development which enhances the exploratory capabilities and field of vision of older infants.

The next key issue we examined was to quantify joint attention episodes across cohorts. Such episodes help shape infant development (Carpenter et al., 1998; Mundy and Newell, 2007; Nelson et al., 2008). We found a similar number of total episodes of joint attention in all cohorts. Again, this consistency across cohorts is remarkable given the differences in cultural contexts. Similarities extended to the proportion of joint attention bouts which were terminated by infants, but not to the episodes initiated by them. UK infants led a greater proportion of joint attention episodes during caregiver-infant interaction relative to the two Indian groups. Nine-month-old Indian infants also led a greater proportion of joint attention episodes than their 6-month-old counterparts. These data may reflect differences in parenting styles across cultures. That is, UK parents are more likely to follow child-centered distal parenting styles and be less directive (Keller, 2007), thus providing greater opportunities for their infants to lead the interactions (Keller et al., 2010). In contrast, the proximal parenting style, which is more common in rural India, is more directive; this is consistent with parents leading a greater proportion of joint attention bouts (Keller et al., 2010). These apparent cultural differences are further nuanced by the infants' age. Perhaps, greater mobility and cognitive skills allow the 9-month-old infants in India to create more opportunities for them to lead joint attention episodes.

After quantifying joint attention episodes, we could then examine infant MLDs across three key contexts: when infants were exploring objects “alone” vs. when they were exploring objects during infant-led and caregiver-led joint attention episodes. Here we found large overall differences in infant MLDs across contexts with shorter MLDs during “looking alone” episodes, longer MLDs in caregiver-led episodes, and even longer MLDs in infant-led episodes. Again, the consistency in this effect across cohorts was striking. One could imagine, for instance, that a more directive parenting style in India might foster longer MLDs in the more “typical” context. This was not the case; rather, all infants showed longer MLDs in infant-led joint attention episodes. These data are consistent with proposals that joint attention episodes are effective at lengthening bouts of visual attention (Yu and Smith, 2017) as parents highlight objects by holding them, labeling objects, and so on. Infant-led bouts may be particularly long because such bouts do not require infants to shift their current focus of attention. Critically, increasing MLDs through joint attention may have important consequences for cognition. For instance, Perone and Spencer (2013) used a neural process model to show how joint attention episodes could lengthen bouts of sustained attention on objects leading to stronger working memory and long-term memory representations of object features. Thus, a key future question is whether infants who experience more joint attention episodes have better visual working memory.

Considering the context of attention (i.e., joint attention vs. “looking alone”) also revealed group differences. When infants directed their attention at toys, 9-month-old infants consistently showed longer MLD than the other two groups regardless of whether they were attending on their own, during infant-led joint attention, or during caregiver-led joint attention. This fits long-standing research on the developmental trajectory of infant visual cognition in the context of coordinated attention in interactions with objects (Bakeman and Adamson, 1984). Among the 6-month-old cohorts, MLD across all contexts was greater for the British infants. This may suggest more advanced visual cognitive dynamics in these infants with longer bouts of sustained attention to toys.

Regarding attention toward the caregiver's face, the 6-month-old infants from India consistently had longer MLD regardless of whether they were attending to the target on their own or in joint attention episodes. This may be partly explained by how they were positioned and by their mobility (Soska and Adolph, 2014; Fausey et al., 2016). Previous research has indicated that the infant-perspective field of view changes with development due to changes in their motor abilities, skills, as well as caretaking needs (Fausey et al., 2016; Jayaraman et al., 2017). Not only do younger infants have more input from faces in their daily lives (Fausey et al., 2016), but they also have fewer opportunities to manually and visually explore objects when in supine and prone positions (Soska and Adolph, 2014). In the case of our findings, the younger Indian infants tended to be placed on their backs, directly in front of their parents. Furthermore, caregivers directed the interaction, moving the toys in and out of the infants' field of view. For the older Indian infants, by contrast, their greater motor and cognitive abilities allowed them to explore their surroundings in a more proactive way.

The UK infants and the older Indian infants displayed similar MLD toward their caregiver's face when looking on their own but displayed interesting differences during bouts of joint attention. When joint attention was led by infants, British infants sustained their attention on their caregivers' faces for longer; however, when joint attention was led by caregivers, the older Indian infants sustained looks toward their caregiver's face for longer than the British infants. These findings may be consistent with cultural differences in parenting styles. In particular, the 9-month-old Indian infants are likely to have experienced more directive parenting whereas the British infants may be more accustomed to child-centered interactions (Lancy, 2014). Each group, thus, engages with their parents differently, with Indian infants being more responsive to faces during caregiver-led attention and British infants being more responsive to faces during infant-led episodes.

Regarding the caregiver's deployment of visual attention, UK caregivers displayed shorter MLD toward toys across all contexts of attention and toward the face of their infant in both joint attention contexts. As mentioned previously, this may reflect either shorter bouts of sustained attention or faster visual processing speed. When looking at the Indian groups, differences in MLD only appeared when the targets were toys and during joint attention episodes led by the caregiver. That is, caregivers of the 9-month cohort displayed shorter MLD directed at toys when they led the joint attention episodes but deployed their visual attention comparatively to the caregivers of the younger cohort in all other cases.

One key limitation in this study was in the differential settings across cultures which may have impacted the switch rate measure. As noted, British dyads completed the play session in their own homes, while Indian dyads completed the play session in a controlled environment. This difference was a consequence of the limited access to the home environment for participants in rural India. Future research could go into homes in rural India. While an attractive option, this would create logistic hurdles as all devices would need battery power and have to be charged in-between sessions. We also suspect the availability of consistent lighting might impact the video recordings.

Another limitation was the lack of information about toy preferences across cultures. It is possible that infants had differential interest in the toys. Although steps were taken to use objects/toys that were appropriate for each culture, including a controlled measure of toy preferences would be valuable for future research. We also note that interactions were being recorded with participants consistently viewing the head-mounted cameras. This might have influenced participants behaviors. As Schmidt et al. (2023) note, patterns of social desirability in behavior should be aligned with the social and cultural norms of the settings. When they are not, this could enhance cultural differences.

In conclusion, the present study developed and deployed a new visual cognition pipeline to reveal insights into visual exploration during dyadic interactions in infancy across socio-cultural settings. Results indicate consistent patterns in caregiver and infants' visual exploratory behavior such that infants from the UK tend to initiate a higher proportion of joint attention episodes compared to the other groups. This fits the eco-cultural model of parenting wherein caregivers in western middle-class families tend to take a child-centric approach (Keller et al., 2005; Keller, 2017). In comparison, infants in India, particularly the younger cohort, tend to lead fewer joint attention episodes. Despite these cultural differences, we also found remarkable consistency in visual exploratory patterns across cultures with, for instance, longer mean look durations during infant-led joint attention episodes relative to caregiver-led episodes and relative to the “looking alone” context. We have demonstrated how the pipeline can be deployed across cultural contexts and with a large number of dyads without the need for time-consuming detailed video coding. Thus, we contend that this pipeline opens up new avenues for exploring how dyadic interactions in infancy help shape development in both high and low resource contexts.

## Data availability statement

The raw data supporting the conclusions of this article will be made available by the authors, without undue reservation.

## Ethics statement

All procedures used in the UK study were reviewed and approved by the UK NHS Health Research Authority Ethics Committee [IRAS (Integrated Research and Application System) ID 196063]. Approval for the India study was provided by the Institutional Ethics Committee at the Community Empowerment Lab (CELIEC/2017002), Lucknow, India. The studies were conducted in accordance with the local legislation and institutional requirements. Written informed consent for participation in this study was provided by the participants' legal guardians/next of kin. Written informed consent was obtained from the individual(s), and minor(s)' legal guardian/next of kin, for the publication of any potentially identifiable images or data included in this article.

## Author contributions

PA: Writing—review & editing, Writing—original draft, Visualization, Validation, Supervision, Methodology, Investigation, Formal analysis, Data curation, Conceptualization. TK: Writing—review & editing, Visualization, Validation, Methodology, Formal analysis. JN: Writing—review & editing, Visualization, Validation, Methodology, Formal analysis. SS: Writing—review & editing, Supervision, Methodology. JC: Writing—review & editing, Supervision, Investigation, Data curation. JM: Writing—review & editing, Supervision, Investigation, Data curation. MT: Writing—review & editing. AK: Writing—review & editing, Funding acquisition. JPS: Writing—review & editing, Supervision, Resources, Project administration, Methodology, Funding acquisition, Formal analysis, Data curation, Conceptualization.

## References

[B1] Abels M. (2020). Triadic interaction and gestural communication: hierarchical and child-centered interactions of rural and urban Gujarati (Indian) caregivers and 9-month-old infants. Dev. Psychol. 56, 1817–1828. 10.1037/dev0001094 32700949

[B2] Abels M. Papaligoura Z. Lamm B. Yovsi R. D. (2017). How usual is“Play as you usually would”? A comparison of naturalistic mother-infant interactions with videorecorded play sessions in three cultural communities. Child Dev. Res. 2017, 1–8. 10.1155/2017/7842030

[B3] Abney D. H. Smith L. B. Yu C. (2017). “It's time: quantifying the relevant timescales for joint attention,” in CogSci.

[B4] Aslin R. N. (2009). How Infants view natural scenes gathered from a head-mounted camera. Optomet. Vision Sci. 86, 561–565. 10.1097/OPX.0b013e3181a76e96 19417702 PMC2748119

[B5] Bakeman R. Adamson L. B. (1984). Coordinating attention to people and objects in mother-infant and peer-infant interaction. Child Dev. 55:1278. 10.2307/1129997 6488956

[B6] Bard K. A. Keller H. Ross K. M. Hewlett B. Butler L. Boysen S. T.. (2021). Joint attention in human and chimpanzee infants in varied socio ecological contexts. Monogr. Soc. Res. Child Dev. 86, 7–217. 10.1111/mono.12435 35355281

[B7] Boukerche A. Hou Z. (2022). Object detection using deep learning methods in traffic scenarios. ACM Comput. Surv. 54, 1–35. 10.1145/3434398

[B8] Brooks R. Singleton J. L. Meltzoff A. N. (2020). Enhanced gaze following behavior in Deaf infants of Deaf parents. Dev. Sci. 23:e12900. 10.1111/desc.12900 31486168 PMC7028450

[B9] Carpenter M. Nagell K. Tomasello M. Butterworth G. Moore C. (1998). Social cognition, joint attention, and communicative competence from 9 to 15 months of age. Monogr. Soc. Res. Child Dev. 63, i–174. 10.2307/1166214 9835078

[B10] Colombo J. Mitchell D. W. Coldren J. T. Freeseman L. J. (1991). Individual differences in infant visual attention: are short lookers faster processors or feature processors? Child Dev. 62, 1247–1257. 10.2307/1130804 1786713

[B11] Delacre M. Lakens D. Leys C. (2017). Why psychologists should by default use welch's t-test instead of student's t-test. Int. Rev. Soc. Psychol. 30, 92–101. 10.5334/irsp.82

[B12] Evans C. A. Porter C. L. (2009). The emergence of mother infant co-regulation during the first year: links to infants' developmental status and attachment. Infant Behav. Dev. 32, 147–158. 10.1016/j.infbeh.2008.12.005 19200603

[B13] Fausey C. M. Jayaraman S. Smith L. B. (2016). From faces to hands: changing visual input in the first two years. Cognition 152, 101–107. 10.1016/j.cognition.2016.03.005 27043744 PMC4856551

[B14] Franchak J. M. Kretch K. S. Soska K. C. Adolph K. E. (2011). Head-mounted eye tracking: a new method to describe infant looking: head-mounted eye tracking. Child Dev.82, 1738–1750. 10.1111/j.1467-8624.2011.01670.x 22023310 PMC3218200

[B15] Friard O. Gamba M. (2016). BORIS: a free, versatile open source event logging software for video/audio coding and live observations. Methods Ecol. Evol. 7, 1325–1330. 10.1111/2041-210X.12584

[B16] Gibbons J. D. Chakraborti S. (2011). “Nonparametric statistical inference,” in International Encyclopedia of Statistical Science, ed. M. Lovric (Berlin, Heidelberg: Springer), 977–979. 10.1007/978-3-642-04898-2_420

[B17] Henrich J. Heine S. J. Norenzayan A. (2010). The weirdest people in the world? Behav. Brain Sci. 33, 61–83. 10.1017/S0140525X0999152X 20550733

[B18] Jayaraman S. Fausey C. M. Smith L. B. (2017). Why are faces denser in the visual experiences of younger than older infants? Dev. Psychol. 53, 38–49. 10.1037/dev0000230 28026190 PMC5271576

[B19] Jocher G. Chaurasia A. Stoken A. Borovec J. Kwon Y. Xie F.. (2022). ultralytics/yolov5: v6.1 - *TensorRT, TensorFlow Edge TPU and OpenVINO Export and Inference*. Genàve: Zenodo

[B20] Kassner M. Patera W. Bulling A. (2014). “Pupil: an open source platform for pervasive eye tracking and mobile gaze-based interaction,” in Proceedings of the 2014 ACM International Joint Conference on Pervasive and Ubiquitous Computing: Adjunct Publication (Seattle Washington: ACM), 1151–1160. 10.1145/2638728.2641695

[B21] Keller H. (2007). Cultures of Infancy. Mahwah, NJ, US: Lawrence Erlbaum Associates Publishers.

[B22] Keller H. (2017). Culture and development: a systematic relationship. Perspect. Psychol. Sci. 12, 833–840. 10.1177/1745691617704097 28972842

[B23] Keller H. Abels M. Lamm B. Yovsi R. D. Voelker S. Lakhani A. (2005). Ecocultural effects on early infant care: a study in Cameroon, India, and Germany. Ethos 33, 512–541. 10.1525/eth.2005.33.4.512

[B24] Keller H. Borke J. Chaudhary N. Lamm B. Kleis A. (2010). Continuity in parenting strategies: a cross-cultural comparison. J. Cross-Cult. Psychol. 41, 391–409. 10.1177/0022022109359690

[B25] Kerby D. S. (2014). The simple difference formula: an approach to teaching nonparametric correlation. Compr. Psychol. 3:11.IT.3.1. 10.2466/11.IT.3.114966997

[B26] Lancy D. F. (2007). Accounting for variability in mother? Child play. Am. Anthropol. 109, 273–284. 10.1525/aa.2007.109.2.273

[B27] Lancy D. F. (2010). Learning ‘from nobody': the limited role of teaching in folk models of children's development. Childhood Past 3, 79–106. 10.1179/cip.2010.3.1.79

[B28] Lancy D. F. (2014). The Anthropology of Childhood: Cherubs, Chattel, Changelings. Cambridge: Cambridge University Press. 10.1017/CBO9781139680530

[B29] Long B. L. Sanchez A. Kraus A. M. Agrawal K. Frank M. C. (2022). Automated detections reveal the social information in the changing infant view. Child Dev. 93, 101–116. 10.1111/cdev.13648 34787894

[B30] Masmoudi M. Friji H. Ghazzai H. Massoud Y. (2021). A reinforcement learning framework for video frame-based autonomous car-following. IEEE Open J. Intell. Transp. Syst. 2, 111–127. 10.1109/OJITS.2021.3083201

[B31] Mundy P. Gomes A. (1998). Individual differences in joint attention skill development in the second year. Infant Behav. Dev. 21, 469–482. 10.1016/S0163-6383(98)90020-0

[B32] Mundy P. Newell L. (2007). Attention, joint attention, and social cognition. Curr. Direct. Psychol. Sci. 16, 269–274. 10.1111/j.1467-8721.2007.00518.x 19343102 PMC2663908

[B33] Nelson P. B. Adamson L. B. Bakeman R. (2008). Toddlers' joint engagement experience facilitates preschoolers' acquisition of theory of mind. Dev. Sci. 11, 847–852. 10.1111/j.1467-7687.2008.00733.x 19046153 PMC2640940

[B34] Padilla R. Passos W. L. Dias T. L. B. Netto S. L. da Silva E. A. B. (2021). A comparative analysis of object detection metrics with a companion open-source toolkit. Electronics 10:279. 10.3390/electronics10030279

[B35] Perone S. Spencer J. P. (2013). Autonomous visual exploration creates developmental change in familiarity and novelty seeking behaviors. Front. Psychol. 4:648. 10.3389/fpsyg.2013.00648 24065948 PMC3778377

[B36] Pouyanfar S. Sadiq S. Yan Y. Tian H. Tao Y. Reyes M. P.. (2019). A Survey on deep learning: algorithms, techniques, and applications. ACM Comput. Surv. 51, 1–36. 10.1145/3234150

[B37] Redmon J. Divvala S. Girshick R. Farhadi A. (2016). “You only look once: unified, real-time object detection,” in 2016 IEEE Conference on Computer Vision and Pattern Recognition (CVPR) (Las Vegas, NV, USA: IEEE), 779–788. 10.1109/CVPR.2016.91

[B38] Rose S. A. Feldman J. F. Jankowski J. J. (2001). Attention and recognition memory in the 1st year of life: a longitudinal study of preterm and full-term infants. Dev. Psychol. 37, 135–151. 10.1037/0012-1649.37.1.135 11206428

[B39] Rose S. A. Feldman J. F. Jankowski J. J. (2002). Processing speed in the 1st year of life: a longitudinal study of preterm and full-term infants. Dev. Psychol. 38, 895–902. 10.1037/0012-1649.38.6.895 12428702

[B40] Schmidt W. J. Keller H. Rosabal Coto M. (2023). The cultural specificity of parent-infant interaction: perspectives of urban middle-class and rural indigenous families in Costa Rica. Infant Behav. Dev. 70:101796. 10.1016/j.infbeh.2022.101796 36410058

[B41] Schneiberg S. Sveistrup H. McFadyen B. McKinley P. Levin M. F. (2002). The development of coordination for reach-to-grasp movements in children. Exper. Brain Res. 146, 142–154. 10.1007/s00221-002-1156-z 12195516

[B42] Schneider C. A. Rasband W. S. Eliceiri K. W. (2012). NIH Image to ImageJ: 25 years of image analysis. Nat. Methods 9, 671–675. 10.1038/nmeth.2089 22930834 PMC5554542

[B43] Siposova B. Carpenter M. (2019). A new look at joint attention and common knowledge. Cognition 189, 260–274. 10.1016/j.cognition.2019.03.019 31015079

[B44] Smith L. B. Yu C. Pereira A. F. (2011). Not your mother's view: the dynamics of toddler visual experience: dynamics of toddler visual experience. Dev. Sci. 14, 9–17. 10.1111/j.1467-7687.2009.00947.x 21159083 PMC3050020

[B45] Smith L. B. Yu C. Yoshida H. Fausey C. M. (2015). Contributions of head-mounted cameras to studying the visual environments of infants and young children. J. Cogn. Dev. 16, 407–419. 10.1080/15248372.2014.933430 26257584 PMC4527180

[B46] Soska K. C. Adolph K. E. (2014). Postural position constrains multimodal object exploration in infants. Infancy 19, 138–161. 10.1111/infa.12039 24639621 PMC3951720

[B47] Sullivan G. M. Feinn R. (2012). Using effect size or why the *p* value is not enough. J. Graduate Med. Educ. 4, 279–282. 10.4300/JGME-D-12-00156.1 23997866 PMC3444174

[B48] Tamis-LeMonda C. S. Shannon J. D. Cabrera N. J. Lamb M. E. (2004). Fathers and mothers at play with their 2- and 3-year-olds: contributions to language and cognitive development. Child Dev. 75, 1806–1820. 10.1111/j.1467-8624.2004.00818.x 15566381

[B49] Tzutalin (2015). LabelImg. Published: Free Software: MIT License.

[B50] Xu T. Chen Y. Smith L. (2011). “It's the child's body: the role of toddler and parent in selecting toddler's visual experience,” in 2011 IEEE International Conference on Development and Learning (ICDL) (IEEE), 1–6.

[B51] Yoshida H. Smith L. B. (2008). What's in view for toddlers? Using a head camera to study visual experience. Infancy 13, 229–248. 10.1080/15250000802004437 20585411 PMC2888512

[B52] Yu C. Smith L. (2016). The social origins of sustained attention in one-year-old human infants. Curr. Biol. 26, 1235–1240. 10.1016/j.cub.2016.03.026 27133869 PMC5387765

[B53] Yu C. Smith L. B. (2012). Embodied attention and word learning by toddlers. Cognition 125, 244–262. 10.1016/j.cognition.2012.06.016 22878116 PMC3829203

[B54] Yu C. Smith L. B. (2013). Joint attention without gaze following: human infants and their parents coordinate visual attention to objects through eye-hand coordination. PLoS ONE 8:e79659. 10.1371/journal.pone.0079659 24236151 PMC3827436

[B55] Yu C. Smith L. B. (2017). Handeye coordination predicts joint attention. Child Dev. 88, 2060–2078. 10.1111/cdev.12730 28186339 PMC6894731

[B56] Yu C. Suanda S. H. Smith L. B. (2019). Infant sustained attention but not joint attention to objects at 9 months predicts vocabulary at 12 and 15 months. Dev. Sci. 22:e12735. 10.1111/desc.12735 30255968 PMC6918481

[B57] Yu C. Yurovsky D. Xu T. (2012). Visual data mining: an exploratory approach to analyzing temporal patterns of eye movements: visual data mining of gaze data. Infancy 17, 33–60. 10.1111/j.1532-7078.2011.00095.x 32693505

[B58] Yu K.-H. Beam A. L. Kohane I. S. (2018). Artificial intelligence in healthcare. Nat. Biomed. Eng. 2, 719–731. 10.1038/s41551-018-0305-z 31015651

[B59] Yuan L. Xu T. L. Yu C. Smith L. B. (2019). Sustained visual attention is more than seeing. J. Exper. Child Psychol. 179, 324–336. 10.1016/j.jecp.2018.11.020 30579246 PMC7670876

[B60] Yurovsky D. Smith L. B. Yu C. (2013). Statistical word learning at scale: the baby's view is better. Dev. Sci. 16, 959–966. 10.1111/desc.12036 24118720 PMC4443688

[B61] Zhang F. Bazarevsky V. Vakunov A. Tkachenka A. Sung G. Chang C.-L.. (2020a). MediaPipe hands: on-device real-time hand tracking. arXiv preprint arXiv:2006.10214.

[B62] Zhang K. Zhang Z. Li Z. Qiao Y. (2016). Joint face detection and alignment using multitask cascaded convolutional networks. IEEE Signal Proc. Lett. 23, 1499–1503. 10.1109/LSP.2016.2603342

[B63] Zhang N. Luo J. Gao W. (2020b). “Research on face detection technology based on MTCNN,” in 2020 International Conference on Computer Network, Electronic and Automation (ICCNEA) (Xi'an, China: IEEE), 154–158. 10.1109/ICCNEA50255.2020.00040

[B64] Zuiderveld K. (1994). “Contrast limited adaptive histogram equalization,” in Graphics Gems (Elsevier), 474–485. 10.1016/B978-0-12-336156-1.50061-6

