# Supplementary material for: Leveraging technological advances to assess dyadic visual cognition during infancy in high- and low-resource settings
Source: Front Psychol. 2024 May 30;15:1376552. doi: 10.3389/fpsyg.2024.1376552 (PMC11169819; doi:10.3389/fpsyg.2024.1376552)
Supplement: Supplementary file 1 [file Table_1.DOCX]

Supplementary Material

Leveraging technological advances to assess dyadic visual cognition during infancy in high- and low-resource settings

Prerna Aneja*, Thomas Kinna, Jacob Newman, Saber Sami, Joe Cassidy, Jordan McCarthy, Madhuri Tiwari, Aarti Kumar, and John P. Spencer

*** Correspondence:** Corresponding Author: [p.aneja@uea.ac.uk](mailto:p.aneja@uea.ac.uk) and [j.spencer@uea.ac.uk](mailto:j.spencer@uea.ac.uk)

# Supplementary Figures

**Figure S1.** Visualisation of the coupled data stream and joint attention for each dyad across three cohorts (6 months UK, 6 months India and 9 months India; N = 12) using the TimeVP toolkit. For each cohort, the first row indicates data for infants followed by their parents (second row). Each colour indicates a different object or social partner's face. Dark blue indicates the social partner's face. White spaces indicate looks to non-target objects.


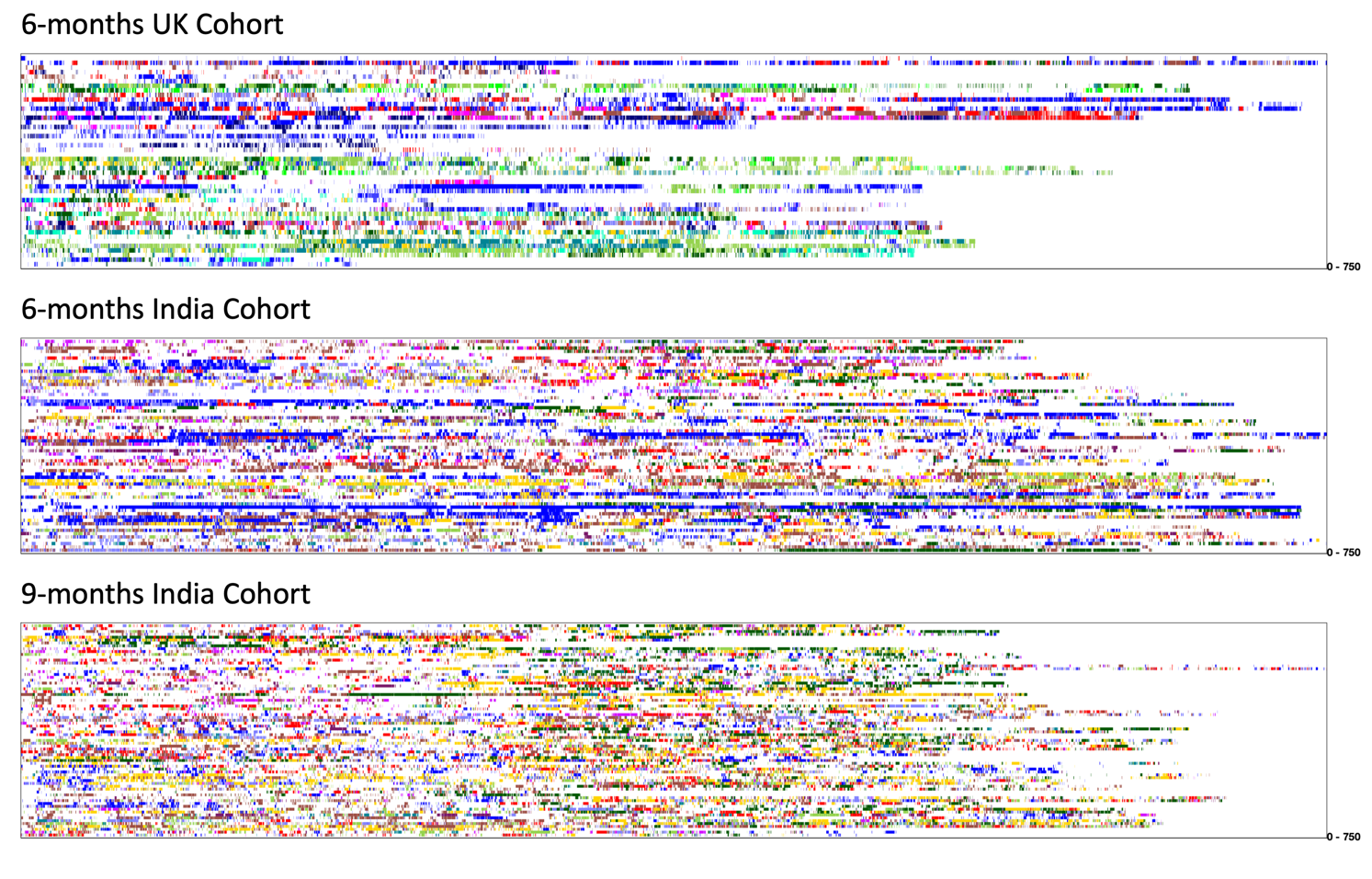


**Figure S2.** Visualisation of the coupled data stream and joint attention for each dyad across three cohorts (6 months UK, 6 months India and 9 months India, N = 94) using the TimeVP toolkit. For each cohort, the first row indicates data for infants followed by their caregivers' (second row). Each colour indicates a different object or social partner's face. Dark blue indicates the social partner's face. White space indicates looking at a non-target.
